# The Complete Chloroplast Genome Sequence of *Laportea bulbifera* (Sieb. et Zucc.) Wedd. and Comparative Analysis with Its Congeneric Species

**DOI:** 10.3390/genes13122230

**Published:** 2022-11-28

**Authors:** Huihui Zhang, Yujing Miao, Xinke Zhang, Guoshuai Zhang, Xiao Sun, Min Zhang, Zhan Feng, Linfang Huang

**Affiliations:** 1 Key Laboratory of Chinese Medicine Resources Conservation, State Administration of Traditional Chinese Medicine of China, Institute of Medicinal Plant Development, Chinese Academy of Medical Sciences, Peking Union Medical College, Beijing 100193, China; 2 School of Pharmacy, Jiangxi University of Chinese Medicine, Nanchang 330000, China

**Keywords:** *Laportea bulbifera*, chloroplast genome, phylogeny, hypervariable regions

## Abstract

*Laportea bulbifera* (*L. bulbifera*) is an important medicinal plant of Chinese ethnic minorities, with high economic and medicinal value. However, the medicinal materials of the genus *Laportea* are prone to be misidentified due to the similar morphological characteristics of the original plants. Thus, it is crucial to discover their molecular marker points and to precisely identify these species for their exploitation and conservation. Here, this study reports detailed information on the complete chloroplast (cp) of *L. bulbifera.* The result indicates that the cp genome of *L. bulbifera* of 150,005 bp contains 126 genes, among them, 37 tRNA genes and 81 protein-coding genes. The analysis of repetition demonstrated that palindromic repeats are more frequent. In the meantime, 39 SSRs were also identified, the majority of which were mononucleotides Adenine-Thymine (A-T). Furthermore, we compared *L. bulbifera* with eight published *Laportea* plastomes, to explore highly polymorphic molecular markers. The analysis identified four hypervariable regions, including *rps*16, *ycf*1, *trn*C-GCA and *trn*G-GCC. According to the phylogenetic analysis, *L. bulbifera* was most closely related to *Laportea canadensis* (*L. canadensis*), and the molecular clock analysis speculated that the species originated from 1.8216 Mya. Overall, this study provides a more comprehensive analysis of the evolution of *L. bulbifera* from the perspective of phylogenetic and intrageneric molecular variation in the genus *Laportea*, which is useful for providing a scientific basis for further identification, taxonomic, and evolutionary studies of the genus.

## 1. Introduction

Chloroplasts (cp) are a unique organelle of green plants, which can participate in photosynthesis and provide the necessary energy for plant growth and development [1]. The chloroplast genome is profoundly conserved, primarily containing genome size, structure, gene content, and organization [2]. It is considered one of the indispensable tools for phylogenetic analysis and molecular identification. At present, numerous studies have utilized plastid information to explore the phylogenetic relationships, origin evolution, and patterns and rates of nucleotide substitutions among land plants [2,3,4]. These studies have demonstrated that angiosperms differ significantly in genome size, genome structure, and gene replacement rates. Consequently, it is also very interesting to understand the plants’ developmental relationship of the same genus through plant plastid data and to analyze their differences from a biological viewpoint.

The genus *Laportea* is an essential group in the Urticaceae family, comprising around 28 species that are mainly distributed in tropical and temperate regions, including Asia, North and South America, Africa, and the Pacific island [5,6,7]. They are mostly perennial herbs, semi-shrub or sparse shrubs, mainly in hillside forests or shrubs [8]. Common features of these plants are alternate leaves, intrapetiolar stipules, and oblique achenes with short stipes [9]. At the same time, most species of *Laportea* show morphological characteristics with four or five male tepals, whereas the female tepals (4 tepals) are extremely uneven. The previous study has illustrated that *Laportea* is perplexed between interspecies and relatives, possibly attributed to the original plant being similar in shape, even though pedicels of female flowers, types of achenes, the leaf margins and the plant’s fluffs have great variation, the profile is very analogous after processing into medicinal materials [10,11]. It also increases the difficulty of identification of herbs inside the genus and the probability of medication blunders, such as *Laportea bulbifera* (*L. bulbifera*), *Laportae aestuans* (*L. aestuans*) and *Laportae violacea* (*L. violacea*) are both used as roots for the treatment of rheumatic pain, antacid activity and skin allergies, respectively, but they are not easily distinguishable in appearance in the difference of leaf margin and tomentum after processing [12,13,14,15,16]. Notably, the genus has high medicinal values, which can cure diabetes, hyperlipidemia, and rheumatoid arthritis, among them, *L. bulbifera* was widely used as an ethnomedicine in China [17].

*L. bulbifera* is a perennial herb, mainly distributed in Guizhou, and is a commonly used medicinal material for the Miao nationality (Miao language medicine name is “*Reib ndad gunb*”). Flavonoids, coumarins, volatile oils, and steroids are the main active ingredients of *L. bulbifera* [18]. Meantime, it has been recorded in “Chinese Materia Medica · Miao Medicine Volume” and “ Quality Standards of Traditional Chinese Medicine and Ethnic Medicine in Guizhou Province". As a traditional Chinese medicine, its mono-formulas or preparations have a good curative effect in the treatment of rheumatic numbness, rheumatoid arthritis, and other diseases [19,20]. However, the current research focuses on the pharmacologically active components of *L. bulbifera*, with fewer reports targeting its genomic aspects. Some studies have only explored the sequencing and assembly of the chloroplast genome of *L. bulbifera*, but have not analyzed the sequence differences, molecular markers, divergence time, or further targeted studies on the phylogenetic evolution and intrageneric variability of the *Laportea* [10]. This has, to some extent, hindered the identification of germplasm resources of *L. bulbifera* and the study of phylogenetic relationships and genetic diversity among the genus *Laportea*.

Therefore, to answer these unresolved questions, the plastid genomes of *L. bulbifera* were sequenced, assembled and annotated, and compared with eight published *Laportea* cp genomes to further explore the genome features and the phylogenetic relationship of *Laportea*. In the meantime, the simple sequence repeats (microsatellites, SSRs) loci and repeat sequence types were identified for investigating the genetic diversity and evaluating the genetic structure; at the same time, the divergence hotspot of *Laportea* was estimated to provide information useful for understanding its evolution and taxonomic identification markers.

## 2. Materials and Methods

### 2.1. Chloroplast DNA Extraction and Sequencing

The leaf material of *L. bulbifera* was gathered from Leigong Mountain, Guizhou, China (26°22′11″ N, 108°10′26″ E) and was identified by Professor Linfang Huang. The samples were deposited in the Herbarium of the Chinese Academy of Medical Science and Peking Union Medicinal College (CMPB14452 to CMPB14454). The fresh leaves were treated with the modified cetyl-trimethyl ammonium bromide (CTAB) technique to isolate the complete genomic DNA [21], and the sequence was performed using paired end on Illumina NovaSeq6000.

### 2.2. Genome Assembly and Annotation

Chloroplast genome annotation was performed using the webserver CPGAVAS2 [22]. To assign each gene, the cutoffs for BLASTn and BLASTx were set to 10^-10^ while manually editing with the Apollo genome editor [23]. We drew the circular gene maps by using an online site (https://www.cloudtutu.com/, accessed on 8 July 2022). In addition, the GC contents of each gene and plastome were calculated by using CGView Serve [24].

### 2.3. Repeat Sequences, Codon Usage and RNA Editing Sites Analysis in L. bulbifera Chloroplast Genome

The SSRs were detected by using MIcroSAtellite (MISA), with parameter settings referenced to Beier et al. [25]. In the meantime, we used the REputer online platform to calculate forward, reverse, palindromic, and complement repeats [26]. In addition, we used Phylosuite v1.2.2 software to extract protein-coding sequence (CDS), and then obtained the relative synonymous codon usage (RSCU) value of *L. bulbifera* by CodonW version1.4.2 calculation [27,28]. The RSCU was calculated as the difference between a codon’s actual and predicted frequency. The codon is utilized less frequently than anticipated if the value of RSCU is lower than 1. Contrarily, it shows that codon use is higher than anticipated [29]. Additionally, the possible RNA editing sites in the CDS of the cp genome were detected by using the predictive RNA Editor for Plants (PREP), with a threshold value of 0.8 [30].

### 2.4. Genome Comparison

To identify interspecific variation, mVISTA was used for plastid comparisons of nine *Laportea* species [31]. Then, sliding window analysis was performed using DnaSP v6.0 to calculate the nucleotide diversity (Pi), with parameter settings referring to the method of Rozas J et al. [31,32]. Finally, the IR boundaries in these genomes were visualized using the IRscope online tool.

### 2.5. Phylogenetic Analysis and the Nucleotide Substitution Rate

In this study, the whole cp genome information for 113 Urticaceae was acquired from the National Center for Biotechnology Information (NCBI) and the sequences of our assembled *L. bulbifera* were merged. In the meantime, *Cannabis sativa* (NC_029855.1), *Humulus lupulus* (NC_028032.1), *Morus alba* (NC_057087.1), *Ficus religiosa (*NC_033979.1), and *Artocarpus camansi* (NC_054247.1) were used as outgroups. Then, 115 CDSs were extracted, aligned, and concatenated using PhyloSuite and MAFFT(v 7.450) [33]. Subsequently, the phylogenetic trees were constructed by RAxML v8.2.4 using maximum likelihood (ML). The detailed parameters were “raxmlHPC-PTHREADS-SSE3-fa -N 1000 –m PROT-GAMMACPREV/GTRGAMMA-x551,314,260-p-551,314,260”. Then, 1000 replicates of the bootstrap analysis were run to determine the significance level of the phylogenetic tree. At the same time, the neighbor-joining (NJ) tree, the test minimum evolution (ME), and the unweighted pair group method with arithmetic mean (UPGMA) tree were constructed by MEGA software, the chosen model Jukes–Cantor, and the bootstrap testing was performed with 1000 repetitions [34,35,36,37]. Similarly, to further explore the affinities within the *Laportea*, we used 78 CDSs to reconstruct the phylogenetic trees. Two species from the *Urticaceae* family, *Debregeasis orientalis* (NC_41413.1), and *Boehmeria umbrosa* (NC_036990.1) were used as an outgroup. Additionally, the nonsynonymous (dN) and synonymous (dS) substitution ratio (dN/dS) of each gene was calculated using the “yn00” program and the F3X4 codon model in PAML v4.9 [38].

### 2.6. Molecular Clock Analyses

The divergence time of *Laportea* was estimated by using BEAST (version 1.10.1) software, with the Bayesian method. The fossil information from *Zhengyia shennongensis* (35 Mya), and *Girardinia suborbiculata* (13 Mya), and the model is an uncorrelated log-normal relaxed clock model [39,40,41]. First, input the combined nuclear genes and chloroplast fragments into BRAUti to generate a “.xml” file that can be imported into BEAST to run, and punctuate the age of the corresponding branch with the age of the fossil. Then, the posterior topology of the tree is set to Yule speciation and runs for 20,000,000 generations to save a tree every 1000 generations. The “trees file” generated by BEAST was imported into TreeAnnotator and the posterior probability limit was set to 0.5 to generate the maximum clade credibility tree (MCC tree) [42]. Finally, the MCC tree was imported into Figuretree (v1.4.3) to show the results.

## 3. Results

### 3.1. Plastome Genomes Structure and Features

**Figure 1:**
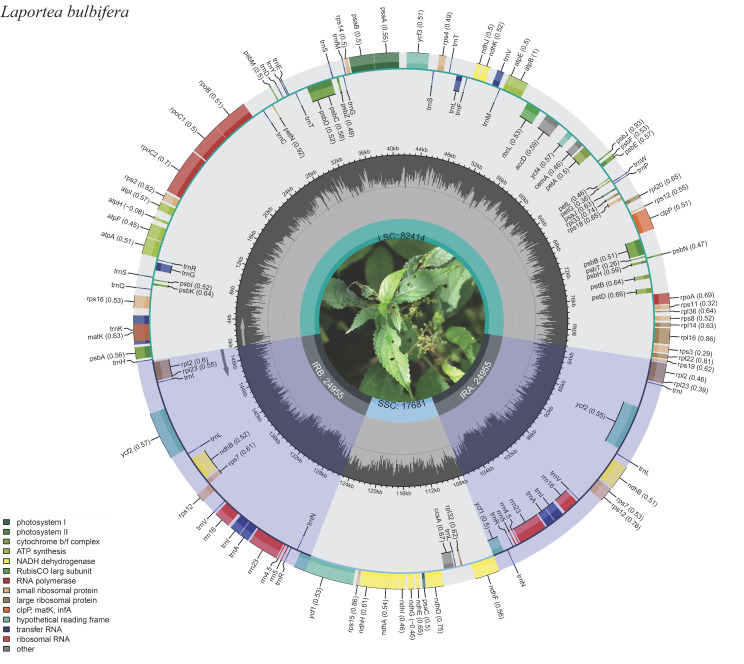
The plastome genome map of *Laportea bulbifera* (*L. bulbifera*) created by CPGAVAS2.

The plastome genome of *L. bulbifer*a was 150,005 bp in length, and display a typical quadripartite circular structure. Among them, a pair of inverted repeats IR regions (IRa and IRb; 24,955 bp), which are isolated by one small single copy (SSC, 17,681 bp) and one large single copy (LSC, 82,414 bp) (Figure 1). The evaluation of the GC content demonstrated that the entire GC content was 36.81%. (Table 1). The IR region, which contained the highest GC content of 42.91%, and the LSC and SSC regions ranked second and third with 34.44% and 30.64%, respectively. (Table 1). In addition, AT content was generally higher than GC content, a characteristic frequently detected in the plastome genomes of angiosperm plants [43,44,45,46,47].

The cp genome of *L. bulbifera* contained 126 different genes, including 81 protein-coding genes (PCGs), 37 transfer RNA (tRNA) genes, and eight ribosomal RNA (rRNA) genes. Among them, seven PCGs (*rps*12, *rps*7, *rpl*23, *rpl*2, *ndh*B, *ycf*1, and *ycf*2), eight tRNA genes (*trn*R-ACG, *trn*A-UGC, *trn*I-GAU, *trn*L-CAA, *trn*I-CAU, *trn*N-GUU, *trn*S-GCU, and *trn*V-GAC), and four rRNA genes (*rrn*16, *rrn*23, *rrn*4.5, and *rrn*5) contained two repeat units (Table 2). In addition, 19 genes are equipped with an intron, among them 11 PCGs (*ndh*A *rps*16, *rpo*C1, *rpl*16, *atp*F, *rpl*2 (×2), *ndh*B (×2), *ycf*1 (×2)) and eight tRNA (*trn*K -UUU, *trn*L-UAA, *trn*V-UAC, *trn*G-UCC, *trn*I-GAU (×2), *trn*A-UGC (×2)) contain only one intron, two protein-coding genes (*ycf*3, *clp*P) contain two introns (Table 3).

### 3.2. Analysis of Repeat Sequences and Codon Usage

**Figure 2:**
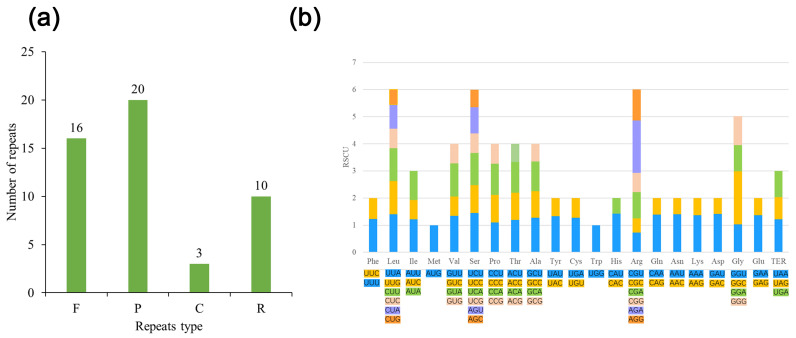
(**a**) Interspersed repeats in *L. bulbifera*, classified by type and frequency (F = forward, P = palindromic, R = reverse and C = complement). (**b**) The 20 amino acids and stop codon of coding genes of the *L. bulbifera* chloroplast genome. The color of the histogram corresponds to the color of codons.

Repeat analysis of *L. bulbifera* plastome detected 39 SSRs with lengths ranging from 17 to 38 bp. It contains 34 mononucleotide repeats (A/T), accounting for 87.20%, and just two polynucleotide repeats (AT). Furthermore, four different forms of interleaved repeats, including 20 palindromic repeats (17–38 bp), 16 forward repeats (18–37 bp), 10 reverse repeats (17–20 bp), and three completion repeats (18 bp) (Figure 2a and Table S1).

The codon count of the *L*. *bulbifera* genome indicated a total of 44,746 codons in protein-coding genes, encoding 20 amino acids (excluding stop codons). Among them Leu encoded the most (4742), accounting for about 10.60%, and Tyr encoded the least (335), accounting for about 0.75% (Table S2). Subsequently, we calculated the codon usage frequency (RSCU values) from the sequence of the protein-coding gene. The result showed that 32 codons with RSCU value greater than 1 and that all except UUG and GGG terminated with A/U (Figure 2b).

In total, 54 RNA editing sites were identified in 12 genes of the *L. bulbifera* cp genome, and *ndh*B was discovered to have the most gene editing sites, while some CDSs only had one editing site (such as *atp*A, *atp*F, *clp*P, and *mat*K, etc.). Furthermore, almost all editing sites underwent a conversion from cytosine to uracil(C-U) at the first or second base position, and no editing sites were discovered at the third codon location. Among these amino acids, the conversion of Serine (S) to leucine (L) is the most frequent. Numerous editing sites have the potential to be transformed, for example, phenylalanine (F), histidine, tyrosine (Y), methionine (M), proline(P), and valine (V) (Table S3).

### 3.3. Comparative Genomic Analysis and Divergence Hotspot Regions

#### 3.3.1. Sliding Window Analysis

**Figure 3:**
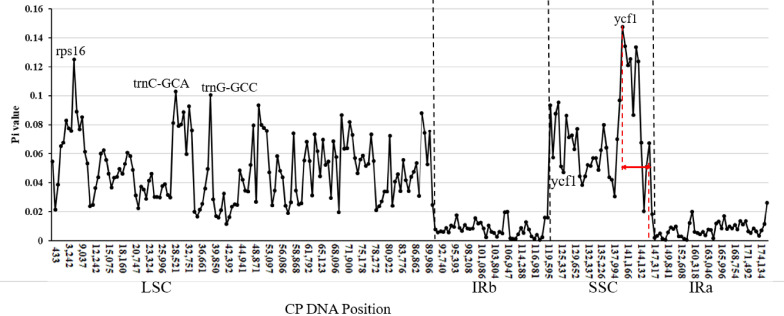
Nucleotide variability (Pi) values among the *Laportea* species. Peak regions with a Pi-value of > 0.10 were tagged with locus tags of genic or intergenic region names. The red arrows show the length range spanned by the protein-coding sequences of *ycf*1.

To better comprehend the genetic diversity, sliding window analysis with the DnaSP programs identified highly variable regions in the *Laportea* cp genome. Figure 3 has elaborated that hotspots of nucleotide divergence four hypervariable regions with Pi values over 0.10, including *rps*16 (Pi = 0.12494), *trn*C-GCA (Pi = 0.10289), *trn*G-GCC (Pi = 0.10067) and *ycf*1 (Pi = 0.12083~0.14722). Noteworthily, the Pi values of the genome in other regions are all greater than 0.02, except for the IR region, demonstrating the *Laportea* cp genome has abundant polymorphisms.

#### 3.3.2. Boundaries of IR

**Figure 4:**
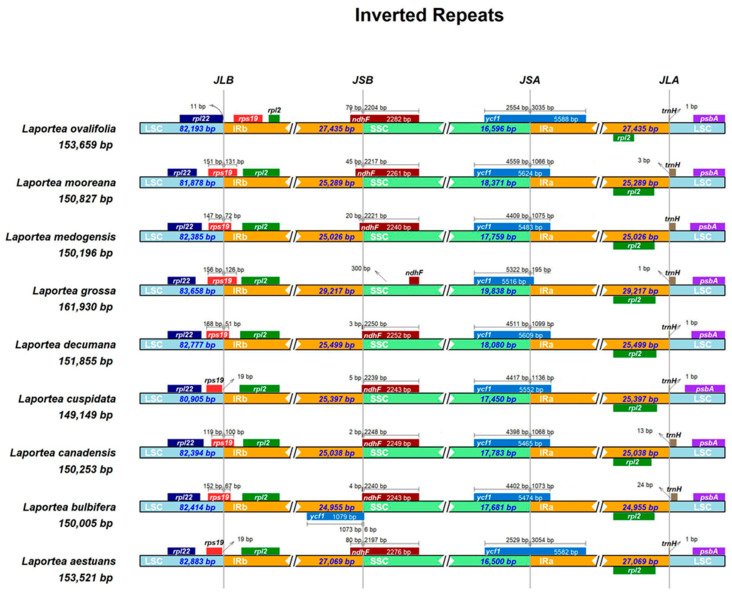
Comparisons of the borders of large single copy (LSC), small singe copy (SSC), and inverted repeats IR regions (IR) among 9 *Laportea* chloroplast genomes.

The expansions and contractions of IR boundaries could reflect the length diversity and evolutionary events in plastid genomes, which are common in cp genomes [48]. Here, we compare differences in size and junction in LSC, SSC, and IR regions of *Laportea*. It indicated that the IR regions’ length varied from 24,955 to 27,435 bp and a plurality of genes spanning or approaching the edges of the IR and SC regions (Figure 4), containing *rps*19, *rpl*2, *rpl*22, *ycf*1, *ndh*F, and *trn*H. The rps19 genes of all species were found in the LSC/IRb border regions, with six bp in the LSC region and 51–131 bp in the IRb region, except for the rps19 genes of *Laportea ovalifolia* (*L. ovalifolia*), *Laportea cuspidate* (*L. cuspidate*), and *L. aestuans*, which were situated in the IRb and LSC regions, respectively. Except that the *ndh*F of *Laportea grossa* is located in the SSC region, other species are located at the junction of the IRb and the SSC region, the SSC region has 2197–2250 bp, and the IRb region has 2–80 bp. The complete *ycf*1 gene is located at the boundary between SSC and IRa, and the length ranges are 2529–5322 bp and 195–3035 bp, respectively. The *trn*H gene is generally situated near the IRb and LSC interface, with a distance to the boundary of 1–24 bp.

#### 3.3.3. Genome Comparison

The mVISTA was used to analyze the sequence variations of *L. bulbifera*, with the annotation of *L. aestuans* as a reference. The results suggest that the majority of PCGs were conserved, several nevertheless exhibited significant change, including *ycf*1, *rpo*C2 and *ccs*A. In addition, intergenic regions such as *trn*S-UGA-*psb*Z-*trnf*M-CAU, *trn*Q-UUG-*trn*S-GCU-*trn*R-UCU, *ycf*3-*trn*S-GGA, *rrn*5-*trn*N-GUU-*ndh*F, *trn*N-GUU-*rrn*4.5S, *pet*A-*pet*L, and *rpl*32-*trn*L-UAG were where the variation was most concentrated (Figure S1). Overall, the IRs regions are more divergent than the LSC and SSC regions.

### 3.4. Gene Selective Pressure Analysis

**Figure 5:**
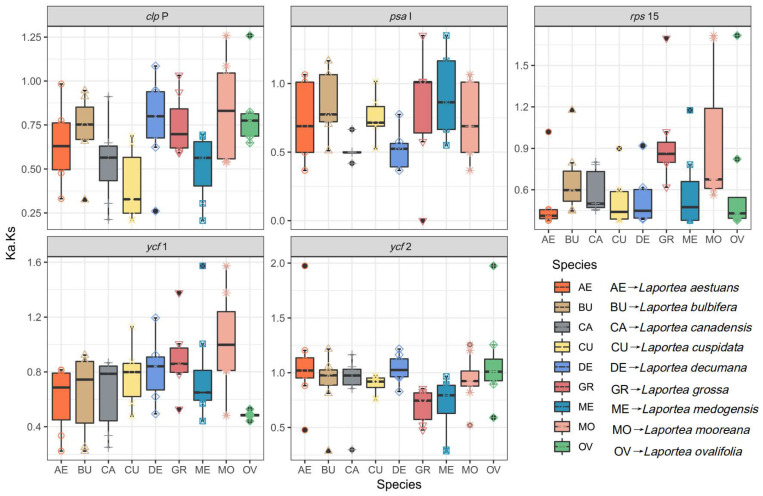
The dN/dS values between each plastid gene in the *Laportea* species are shown as box plots.

The dN/dS ratio could provide insight into the evolution of DNA sequence by examining the process of diversification selection among related species [49,50,51,52,53]. In this study, the majority of the 62 shared genes in this research had dN/dS ratios smaller than 1 (Figure S2), which suggests that they may be in the process of purifying selection. While five genes (*clp*P, *psa*l, *rps*15, *ycf*1, and *ycf*2) have higher dN/dS ratios, ranging from 0.6 to 1.0. It was demonstrated that these genes have some potential for positive gene selection, which may speed up the future development of *Laportea* species (Figure 5). In general, the lower substitution rates of plastids in the *Laportea* indicate that PCGs were highly conserved.

### 3.5. Phylogenetic Analysis and Divergence Time Estimation

**Figure 6:**
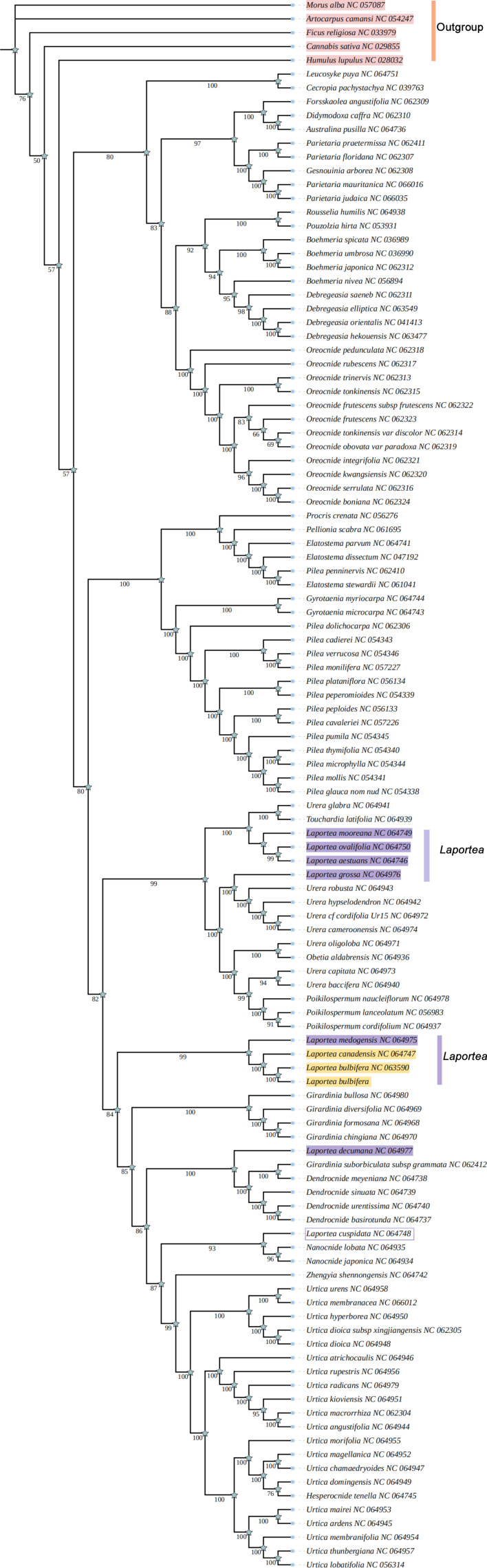
The phylogenetic tree of species from Urticaceae is based on the 115 CDSs from 114 taxa using the maximum likelihood (ML) method. The purple markers are species of the genus *Laportea*. The yellow section shows that both the data from the previous study and the resequencing data in the present study indicate that *L. bulbifera* and *L. canadensis* are most closely related. The purple part indicates species of the genus *Laportea*, *L. decumana* clustered as a single clade, *L. cuspidata* clustered as a clade with species of the genus *Nanocnide*.

**Figure 7:**
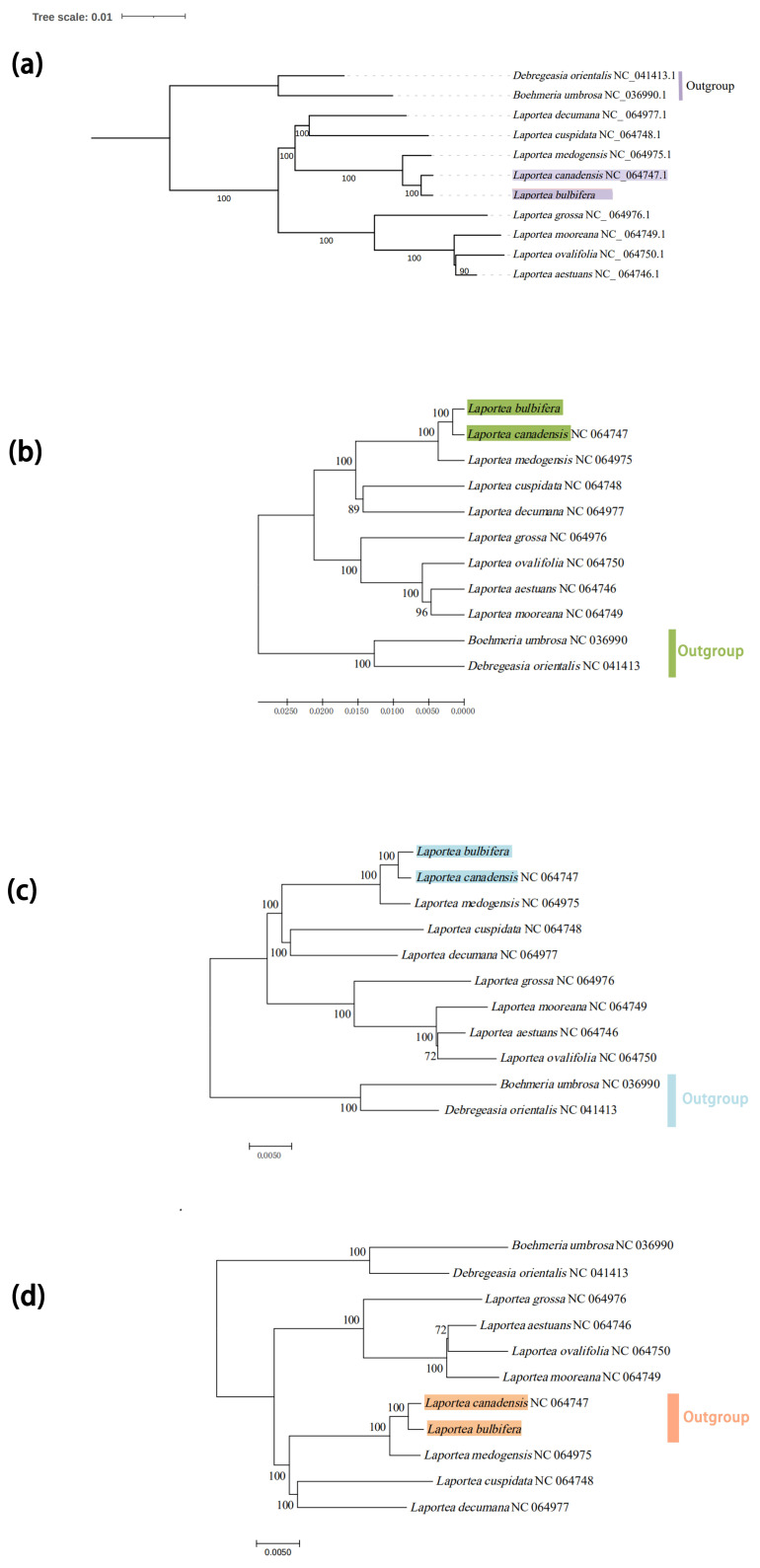
The phylogenetic tree of species from *Laportea* is based on 78 CDSs. (**a**) Phylogenetic tree constructed using the maximum likelihood (ML) methods. (**b**) Phylogenetic tree constructed using the unweighted pair group method with arithmetic mean (UPGMA) methods. (**c**) Phylogenetic tree constructed using the minimum evolution (ME) methods. (**d**) Phylogenetic tree constructed using the neighbor-joining (NJ) methods.

**Figure 8:**
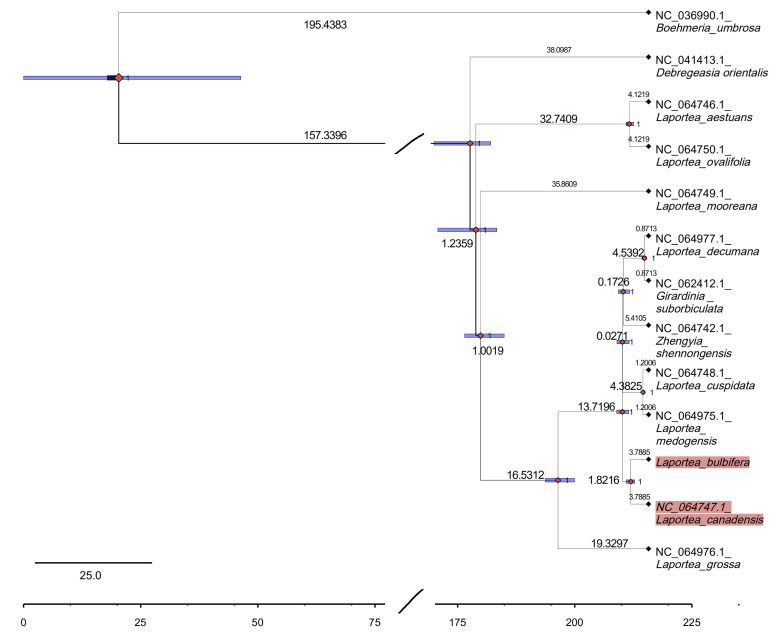
Divergence time estimation. The phylogenetic tree was reconstructed based on 78 CDSs by using the maximum likelihood (ML) methods and the bars show the 95% highest posterior density on the age estimates. The red highlighted part indicates the closest relationship between *L. canadensis* and *L. bulbifera*, and probably splitting at~1.8216 Mya.

Four phylogenetic trees (ML, UPGMA, ME, and NJ) of Urticaceae were constructed based on the 115 CDSs. This result showed that the tree has higher support values on the whole, and the taxonomy of species in the genus *Laportea* is confusing, most of them were found scattered in *Urera*, *Poikilospermum*, *Girardina*, and *Naporea*. Specifically, it was clustered into four to five branches. Among these, both the ML tree and ME tree showed that *L. bubifera*, *L. canandensis*, and *L. medogensis* clustered into one branch, *L. moreana*, *L. ovalifolia*, *L. aistuans,* and *L. grossa* clustered into one branch. And *L. decumana* and *L. cuspidata* clustered as one, respectively, while NJ and UPGMA showed *L. grossa* as a separate species (Figure 6 and Figure S4). The results of the construction of species of Urticaceae in this study are generally consistent with previous studies [10,17]. Subsequently, to explore the affinities within the *Laportea*, we constructed four phylogenetic trees. The result showed that all nodes had the highest bootstrap support, and *Laportea* could be grouped into two clades, one clade includes four species (*L. grossa*, *L. mooreana*, *L. ovalifolia,* and *L. aestuans*), whereas the other clades include the remaining species. Phylogenetic tree analysis at the family and genus level indicated that *L. bulbifera* was closely related to *L.canadensis* (Figure 7). Furthermore, the divergence time of each internal node of the phylogenetic tree was estimated with fossil record data of *Zhengyia shennongensis* and *Girardinia suborbiculata* to calibrated by using BEAST for further infer the historical origin of *Laportea* species. It was shown that *Laportea* genus diverged at about ~157.3396 million years ago (Mya), with *L. bulbifera* splitting at~1.8216 Mya (Figure 8). The detected divergence time may contribute to future research on the *Laportea* genus.

## 4. Discussion

The Urticaceae family is diverse, with approximately 54 genera and ∼2600 species worldwide, and is divided into six groups overall, including Boehmerieae, Cecropiaceae, Elatostemateae, Forsskaoleae, Parietarieae, and Urticeae [54]. Molecular studies at this stage support that Urticaceae constitute a good branch, while the majority of this research has focused on the family or tribe level, with relatively few investigations on congenic species [55]. *Laportea* is a genus of the tribe Urticeae, and phylogenetic analysis demonstrates that *Laportea* consisted of a polylineage scattered in the *Urera* and *Poikilospermum* clade. [8,56]. In this study, we reconstructed the phylogenetic analysis of Urticaceae family, which were consistent with those of previous studies, showing that *Laportea* was mostly dispersed in *Urera*, *Poikilospermum*, *Girardina*, and *Naporea*. However, to date, no studies have targeted the phylogenetic relationships and intra-genus differences of this genus.

Here, we first sequenced, assembled, annotated, and processed data for chloroplasts of *L. bulbifera*. Then, the complete plastid data of eight *Laportea* species were downloaded from NCBI and aligned and concatenated using the MAFFT online website. Subsequently, the genetic differences, high variance regions, and evolutionary history of *Laportea* were analyzed using bioinformatics tools such as mVISTA and IRscope online sites, MEGA, and DnaSP software. The result indicated that the plastomes of *Laportea*, with sizes ranging from 149,149 bp to 161,930 bp, exhibited the tetrad structure typical of angiosperms. The cp genome had an uneven distribution of GC content, with IR regions having a higher abundance than LSC and SSC. The possible reason for this phenomenon is that the IR region is enriched with ribosomal RNA (rRNA) genes and transfer RNA (tRNA) genes of GC. At the same time, the conservatism in IR regions compared to SC regions may also be due to GC inequality [57,58]. In addition, previous studies have demonstrated that changes in IR/SC junctions are thought to be one of the main drivers of the size diversity of cp genomes in higher plants [59,60]. Changes in the length of the cp genome may be mostly caused by the shrinkage and extension of IR border regions [48]. Genes located on the border could make IR or SC sections with the extension or shrinkage of the IR boundary regions. (e.g., rps19, ycf1, and *trn*H shown in Figure 4). The result is consistent with previous findings, both indicating that the cp genomes of species within the same family or genus are extremely homogeneous [17].

The SSRs are a type of genetic marker that reveal information about an individual and are composed of tandem repeats of 1–6 oligonucleotides [61,62]. The SSRs analysis revealed *L. bulbifera* has the highest number of mononucleotides in chloroplasts, most of which were poly T and A. This is consistent with earlier research indicating that mononucleotides are the most abundant type of SSRs and a majority of these loci are located in the noncoding regions, as in most angiosperms [63,64,65,66]. All in all, the SSRs resource established will be beneficial for plant evolution and ecological studies of *Laportea*.

This study was the first opportunity to compare the *Laportea* plastid genome and estimate the ratios of dN/dS by using mVISTA online sites and PAML v4.9 program to reveal the interspecific diversity of plastid genomes in *Laportea*. It was shown that the noncoding regions of the plastids in *Laportea* display higher polymorphisms than the coding regions, which is the same result as most angiosperms [67,68] (Figure 5 and Figure S2). The hypervariability analysis is also consistent with this viewpoint. In the meantime, the result recommends four hypervariable genes, *rps*16, t*rnC*-GCA, *trnG*-GCC, and *ycf*1, as potential molecular markers of the *Laportea*. Subsequently, to verify whether the above-mentioned encoded proteins can be used as molecular markers, we extracted these protein-coding genes and reconstructed the phylogenetic tree to explore their taxonomic relationship. The results are consistent with the previous 78 protein-coding gene construction tree files, and the species of *Laportea* are also clustered into two branches, and *L. bulbifera* and *L. canadensis* also have the closest relationship (Figure S3). Thus, we can reasonably speculate that genes such as *ycf*1, *rpo*C2, and *clp*P may serve as the identification points for the evolution of *Laportea*. This is also consistent with the locus *ycf*1 reported by previous studies in Urticaceae, as a highly variable gene, which has critical implications for our effective identification and wise utilization of medicinal taxa in this genus [69,70]. However, this study only made bold speculation from the perspective of phylogenetic analysis, and the specific molecular experimental verification deserves further discussion by subsequent scholars due to the limitations of the sample. Notably, *L. canadensis* is a clonal, monoecious, perennial herb common in North American wetland and floodplain forests and has now been introduced in most countries [71]. At present, some studies have demonstrated that *L. canadensis* has good efficacy in the treatment of skin diseases as well as *Stinging Nettle* [7]. *L. bulbifera* is used clinically mainly for the treatment of analgesic, anti-inflammatory, and rheumatic diseases. However, the similar morphological characteristics of the two plants and the closest kinship hinder their clinical medicinal identification to some extent. Thus, mining the molecular markers between the two is crucial for solving the problem of medicinal confusion within the genus *Laportea*. There are relatively few studies on the medicinal components and pharmacologically active substances of *Laportea*. Here, we suggest that scholars consider *L. bulbifera* as a pioneer in bioprospecting to further promote the clinical medicinal development of this genus.

The complete cp genome could provide a wealth of resources for phylogenetic and evolutionary connection inference [72]. The phylogenetic relationship of *Laportea* has long been controversial. Most of the previous reports focused on a single cp genome [73,74], or analyzed the phylogeny from the perspective of the entire Urticaceae [17], but there was no specific plastid genome analysis on *Laportea*. In this study, the ethnomedicine *L. bulbifera* was taken as an example to interpret the composition of its cp genome from the perspective of plant evolution, and explore its genetic relationship and taxonomic identification points. The results show that the phylogenetic tree has high consensus support at each node indicating the correctness and confidence of the phylogenetic relationship, which also lays the foundation for resolving the controversial issues of the taxonomic status and evolutionary relationship of *Laportea*.

## 5. Conclusions

To further search for molecular markers to solve the confusion within the genus *Laportea* and further rationalize the exploitation of the ethnomedicine *L. bulbifera*, we resequenced and reported the plastid genome of *L. bulbifera*, reconstructed intra-genus relatedness, counted the codon usage, compared the sequence divergence of this genus, and estimated their evolution time for the first time. Overall, the chloroplast genome of *L. bulbifera* contains 39 SSRs, 54 RNA editing sites, and 44,746 codons. The species of *Laportea* exhibit typical circular tetrads, similar to most angiosperms, with sizes ranging from 149,149 bp to 161,930 bp and the characteristics of the *L. bulbifera* plastids are similar to other species of the *Laportea* genus. The genome comparison revealed rps16, *trn*C-GCA, *trn*G-GCC, and *ycf*1 can be considered as potential molecular markers for *Laportea*. The phylogenetic analyses show that *L. bulbifera* and *L. canadensis* are closer, originating about 1.8216 Mya ago. Moreover, three genes with large differences and eight gene spacer regions were detected, which also laid the theoretical foundation for the identification of *Laportea* plants. This research will provide precious resources for the cp genome of *L. bulbifera*, which also has essential implications for investigating the *Laportea* genus evolution and the discrimination of medicinal products.

## Figures and Tables

**Table 1:** Statistics on the basic features of plastome genome of *L. bulbifera*.

Species		*L. bulbifera*
Length (bp)	Total	150,005
	LSC	82,414
	SSC	17,681
	IR	24,955
GC content (%)	Total	36.81
	LSC	34.44
	SSC	30.64
	IR	42.91
Gene numbers	Total	126
	Protein-coding gene	81
	tRNA gene	37
	rRNA gene	8

**Table 2:** Genes identified in the plastome genome of *L. bubifera*.

Category of Genes	Group of Genes	Name of Genes
rRNA	rRNA gene	*rrn*16s (×2)*, rrn*23s (×2)*, rrn*4.5s (×2)*, rrn*5s (×2)
tRNA	tRNA gene	*trn*R-UCU, *trn*G-UCC, *trn*H-GUG, *trn*K-UUU, *trn*Q-UUG, *trn*C-GCA, *trn*D-GUC, *trn*Y-GUA, *trn*E-UUC, *trn*L-UAG, *trn*T-GGU, *trn*S-UGA, *trn*G-GCC, *trnf*M-CAU, *trn*T-UGU, *trn*L-UAA, *trn*F-GAA, *trn*V-UAC, *trn*M-CAU, *trn*W-CCA, *trn*P-UGG, *trn*I-CAU (×2), *trn*L-CAA (×2), *trn*V-GAC (×2), *trn*I-GAU (×2), *trn*A-UGC (×2), *trn*R-ACG (×2), *trn*N-GUU (×2), *trn*S-GCU (×2)
Self-replication	Large subunit of ribosome	*rpl*14, *rpl*16, *rpl*2(×2), *rpl*20, *rpl*22, *rpl*23(×2), *rpl*32, *rpl*33, *rpl*36
	DNA-dependent RNA polymerase	*rpo*A, *rpo*B, *rpo*C1, *rpo*C2
	Small subunit of ribosome	*rps*11, *rps*12(×2), *rps*14, *rps*15, *rps*16, *rps*18, *rps*19, *rps*2, *rps*3, *rps*4, *rps*7(×2), *rps*8
Photosynthesis	Subunits of ATP synthase	*atp*A*, atp*B*, atp*E*, atp*F*, atp*H*, atp*I
	Subunits of photosystem II	*psb*A*, psb*B, *psb*C*, psb*D*, psb*E*, psb*F*, psb*I*, psb*J*, psb*K*, psb*M*, psb*N*, psb*T*, psb*Z*, ycf*3
	Subunits of NADH-dehydrogenase	*ndh*A*, ndh*B (×2)*, ndh*D*, ndh*E*, ndh*F*, ndh*G*, ndh*H*, ndh*I*, ndh*J, *ndh*K
	Subunits of cytochrome b/f complex	*pet*A*, pet*B*, pet*D*, pet*G*, pet*L*, pet*N
	Subunits of photosystem I	*psa*A*, psa*B*, psa*C*, psa*J
	Subunit of rubisco	*rbc*L
Other genes	Subunit of Acetyl-CoA-carboxylase	*acc*D
	c-type cytochrom synthesis gene	*ccsA*
	Envelop membrane protein	*cem*A
	Protease	*clp*P
	Maturase	*mat*K
Genes of unknown	Conserved open reading frames	*ycf*1 (×2), *ycf*2 (×2)*, ycf*4

**Table 3:** The lengths of introns and exons for the splitting genes.

Gene	Strand			Length (bp)				
		Start	End	ExonI	IntronI	ExonII	IntronII	ExonIII
*trn*K-UUU	—	1613	4232	37	2546	37		
*rps*16	—	4891	6016	42	865	219		
*trn*G-UCC	+	8582	9344	23	692	48		
*atp*F	—	11,364	12,614	145	708	398		
*rpo*C1	—	20,130	22,961	432	753	1647		
*ycf*3	—	41,692	43,696	124	776	232	722	151
*trn*L-UAA	+	46,505	47,065	35	476	50		
*trn*V-UAC	—	49,963	50,587	37	551	37		
*clp*P	—	68,438	70,389	71	746	294	615	226
*rpl*16	—	79,433	80,856	9	1016	399		
*rpl*2	—	82,603	84,102	391	675	434		
*ndh*B	—	92,571	94,788	775	685	758		
*trn*I-GAU	+	99,678	100,648	37	897	37		
*trn*A-UGC	+	100,707	101,573	38	794	35		
*ycf*1	+	106,297	107,376	762	30	288		
*ndh*A	—	116,555	118,805	553	1153	545		
*ycf*1	—	120,649	126,123	762	30	4683		
*trn*A-UGC	—	130,847	131,713	38	794	35		
*trn*I-GAU	—	131,772	132,742	37	897	37		
*ndh*B	+	137,632	139,849	775	685	758		
*rpl*2	+	148,318	149,817	391	675	434		

## Data Availability

No new data were created or analyzed in this study. Data sharing is not applicable to this article.

## Supplementary Materials

The following supporting information can be downloaded at: https://www.mdpi.com/article/10.3390/genes13122230/s1, Table S1. Chloroplast genomes are scattered in repeat sequence characteristic values; Table S2. Codon Usage in this chloroplast genome; Table S3. Predicted RNA editing site in *L.bubifera* chloroplast genome; Figure S1. Sequence alignment of *Laportea* chloroplast genomes performed using the mVISTA program with *L. aestuans* as a reference. Figure S2. The dN/dS values between each plastid gene in the Laportera species are shown as box plots. Figure S3. The phylogenetic tree of species from *Laportea* is based on the nucleotide sequences of 8 CDSs using the maximum likelihood (ML) method. Figure S4. The phylogenetic tree of species from Urticaceae based on 115 CDSs using the test minimum evolution (ME, Figure S4a) and the unweighted pair group method with arithmetic mean (UPGMA, Figure S4b) and the neighbor-joining (NJ, Figure S4c) method.

## References

[1] Raven J.A., Allen J.F. (2003). Genomics and chloroplast evolution: What did cyanobacteria do for plants?. Genome Biol..

[2] Tonti-Filippini J., Nevill P.G., Dixon K., Small I. (2017). What can we do with 1000 plastid genomes?. Plant J..

[3] Allen J.F. (2015). Why chloroplasts and mitochondria retain their own genomes and genetic systems: Colocation for redox regulation of gene expression. Proc. Natl. Acad. Sci. USA.

[4] Barrett C.F., Wicke S., Sass C. (2018). Dense infraspecific sampling reveals rapid and independent trajectories of plastome degradation in a heterotrophic orchid complex. New Phytol..

[5] Chew W.-L. (1969). Discocnide (Urticaceae). Gard. Bull. Singap..

[6] Ferguson D.K. (2004). Flora of China Volume 9: Pittosporaceae through Connaraceae by Zhengyi Wu; Peter H. Raven. Systematic Botany.

[7] Flint R.L., Norton S.A., Strausborger S. (2022). Laportea canadensis, the Canada Wood Nettle: Similar to Stinging Nettle in Appearance, Geographic Distribution, and Pharmacologic Effects on the Skin. Dermatitis.

[8] Wu Z.-Y., Monro A.K., Milne R.I., Wang H., Yi T.-S., Liu J., Li D.-Z. (2013). Molecular phylogeny of the nettle family (Urticaceae) inferred from multiple loci of three genomes and extensive generic sampling. Mol. Phylogenet. Evol..

[9] Boufford D.E. (1992). Urticaceae nettle family. J. Ariz. -Nev. Acad. Sci..

[10] Kim C., Deng T., Chase M., Zhang D.-G., Nie Z.-L., Sun H. (2015). Generic phylogeny and character evolution in Urticeae (Urticaceae) inferred from nuclear and plastid DNA regions. Taxon.

[11] Macdonald A. (2011). Theoretical problems of interpreting floral organogenesis of Laportea canadensis. Can. J. Bot..

[12] Wang J., Lu J., Lan Y., Zhou H., Li W., Xiang M. (2013). Total coumarins from Urtica dentata Hand prevent murine autoimmune diabetes via suppression of the TLR4-signaling pathways. J. Ethnopharmacol..

[13] Chen Y., Zou S., Xu W., Sun Q., Yun L. (2020). Spectrum–effect relationship of antioxidant and anti-inflammatory activities of Laportea bulbifera based on multivariate statistical analysis. Biomedical Chromatography.

[14] Lu X., Zhao Y., Li B., Feng W., Qi J., Feng B. (2022). Phytochemical, Chemotaxonomic and Bioinformatics Study on Laportea bulbifera (Urticaceae). Chem. Biodivers..

[15] Chaniad P., Tewtrakul S., Sudsai T., Langyanai S., Kaewdana K. (2020). Anti-inflammatory, wound healing and antioxidant potential of compounds from Dioscorea bulbifera L. bulbils. PLoS ONE.

[16] Christensen C.B., Soelberg J., Jäger A.K. (2015). Antacid activity of *Laportea aestuans* (L.) Chew. J. Ethnopharmacol..

[17] Ogoma C.A., Liu J., Stull G.W., Wambulwa M.C., Oyebanji O., Milne R.I., Monro A.K., Zhao Y., Li D.-Z., Wu Z.-Y. (2022). Deep Insights Into the Plastome Evolution and Phylogenetic Relationships of the Tribe Urticeae (Family Urticaceae). Front. Plant Sci..

[18] Zhu Z., Ma L., Zhu H., Yang X., Hao X. (2011). Studies on the chemical constituents of *Laportea bulbifera*. J. Chin. Med. Mater..

[19] Qiu Dewen D.J. (2005). Chinese Materia Medica Miao Medicine Volume.

[20] Guizhou Medical Products Administration (2020). Quality Standards of Traditional Chinese Medicine and Ethnic Medicine in Guizhou Province.

[21] Arseneau J.R., Steeves R., Laflamme M. (2017). Modified low-salt CTAB extraction of high-quality DNA from contaminant-rich tissues. Mol. Ecol. Resour..

[22] Shi L., Chen H., Jiang M., Wang L., Wu X., Huang L., Liu C. (2019). CPGAVAS2, an integrated plastome sequence annotator and analyzer. Nucleic Acids Res..

[23] Lewis S.E., Searle S., Harris N., Gibson M., Iyer V., Richter J., Wiel C., Bayraktaroglu L., Birney E., Crosby M. (2002). Apollo: A sequence annotation editor. Genome Biol..

[24] Grant J.R., Stothard P. (2008). The CGView Server: A comparative genomics tool for circular genomes. Nucleic Acids Res..

[25] Beier S., Thiel T., Münch T., Scholz U., Mascher M. (2017). MISA-web: A web server for microsatellite prediction. Bioinformatics.

[26] Kurtz S., Choudhuri J.V., Ohlebusch E., Schleiermacher C., Stoye J., Giegerich R. (2001). REPuter: The manifold applications of repeat analysis on a genomic scale. Nucleic Acids Res..

[27] Peden J. (2005). CodonW, version 1.4.2.

[28] Peden J. (1999). Analysis of Codon Usage. Ph.D. Thesis.

[29] Sharp P.M., Li W.-H. (1986). Codon usage in regulatory genes in Escherichia coli does not reflect selection for ‘rare’codons. Nucleic Acids Res..

[30] Mower J.P. (2009). The PREP suite: Predictive RNA editors for plant mitochondrial genes, chloroplast genes and user-defined alignments. Nucleic Acids Res..

[31] Frazer K.A., Pachter L., Poliakov A., Rubin E.M., Dubchak I. (2004). VISTA: Computational tools for comparative genomics. Nucleic Acids Res..

[32] Rozas J., Ferrer-Mata A., Sánchez-DelBarrio J.C., Guirao-Rico S., Librado P., Ramos-Onsins S.E., Sánchez-Gracia A. (2017). DnaSP 6: DNA sequence polymorphism analysis of large data sets. Mol. Biol. Evol..

[33] Rozewicki J., Li S., Amada K.M., Standley D.M., Katoh K. (2019). MAFFT-DASH: Integrated protein sequence and structural alignment. Nucleic Acids Res..

[34] Rzhetsky A., Nei M. (1991). A Simple Method for Estimating and Testing Minimum-Evolution Trees. Mol. Biol. Evol..

[35] Som A. (2006). Theoretical foundation to estimate the relative efficiencies of the Jukes-Cantor+gamma model and the Jukes-Cantor model in obtaining the correct phylogenetic tree. Gene.

[36] Khan H.A., Arif I.A., Bahkali A.H., Al Farhan A.H., Al Homaidan A.A. (2008). Bayesian, maximum parsimony and UPGMA models for inferring the phylogenies of antelopes using mitochondrial markers. Evol. Bioinform. Online.

[37] Hall B.G. (2013). Building phylogenetic trees from molecular data with MEGA. Mol. Biol. Evol..

[38] Yang Z. (2007). PAML 4: Phylogenetic analysis by maximum likelihood. Mol. Biol. Evol..

[39] Takhtajan A. (1982). Ulmaceae-Betulaceae. Foss. Flower. Plants USSR.

[40] Deng T., Kim C., Zhang D.-G., Zhang J.-W., Li Z.-M., Nie Z.-L., Sun H. (2013). Zhengyia shennongensis: A new bulbiliferous genus and species of the nettle family (Urticaceae) from central China exhibiting parallel evolution of the bulbil trait. Taxon.

[41] Bouckaert R., Vaughan T.G., Barido-Sottani J., Duchêne S., Fourment M., Gavryushkina A., Heled J., Jones G., Kühnert D., De Maio N. (2019). BEAST 2.5: An advanced software platform for Bayesian evolutionary analysis. PLoS Comput. Biol..

[42] Bouckaert R., Heled J., Kühnert D., Vaughan T., Wu C.-H., Xie D., Suchard M.A., Rambaut A., Drummond A.J. (2014). BEAST 2: A software platform for Bayesian evolutionary analysis. PLoS Comput. Biol..

[43] Zhang T., Fang Y., Wang X., Deng X., Zhang X., Hu S., Yu J. (2012). The complete chloroplast and mitochondrial genome sequences of Boea hygrometrica: Insights into the evolution of plant organellar genomes. PLoS ONE.

[44] Zhang X., Miao Y., Sun X., Jiang Y., Zeng T., Zheng Y., Huang L. (2022). The complete chloroplast genome sequence of Gentiana triflora and comparative analysis with its congeneric species. J. Appl. Bot. Food Qual..

[45] Zhang X., Zhou T., Kanwal N., Zhao Y., Bai G., Zhao G. (2017). Completion of Eight Gynostemma BL. (Cucurbitaceae) Chloroplast Genomes: Characterization, Comparative Analysis, and Phylogenetic Relationships. Front. Plant Sci..

[46] Zeng S., Zhou T., Han K., Yang Y., Zhao J., Liu Z.L. (2017). The Complete Chloroplast Genome Sequences of Six Rehmannia Species. Genes.

[47] Wang W., Yang T., Wang H.L., Li Z.J., Ni J.W., Su S., Xu X.Q. (2020). Comparative and Phylogenetic Analyses of the Complete Chloroplast Genomes of Six Almond Species (*Prunus* spp. L.). Sci. Rep..

[48] Goulding S.E., Wolfe K., Olmstead R., Morden C. (1996). Ebb and flow of the chloroplast inverted repeat. Mol. Gen. Genet. MGG.

[49] Duchene D., Bromham L. (2013). Rates of molecular evolution and diversification in plants: Chloroplast substitution rates correlate with species-richness in the Proteaceae. BMC Evol. Biol..

[50] Miao Y., Chen H., Xu W., Yang Q., Liu C., Huang L. (2022). Structural mutations of small single copy (SSC) region in the plastid genomes of five Cistanche species and inter-species identification. BMC Plant Biol..

[51] Hjelmen C.E., Johnston J.S. (2017). The mode and tempo of genome size evolution in the subgenus Sophophora. PLoS ONE.

[52] Banerjee R., Chaudhari N.M., Lahiri A., Gautam A., Bhowmik D., Dutta C., Chattopadhyay S., Huson D.H., Paul S. (2021). Interplay of Various Evolutionary Modes in Genome Diversification and Adaptive Evolution of the Family Sulfolobaceae. Front. Microbiol..

[53] Wilson D.J. (2020). GenomegaMap: Within-Species Genome-Wide dN/dS Estimation from over 10,000 Genomes. Mol. Biol. Evol..

[54] Conn B.J., Hadiah J.T. (2009). Nomenclature of tribes within the Urticaceae. Kew Bull..

[55] Sytsma K.J., Morawetz J., Pires J.C., Nepokroeff M., Conti E., Zjhra M., Hall J.C., Chase M.W. (2002). Urticalean rosids: Circumscription, rosid ancestry, and phylogenetics based on rbcL, trnL-F, and ndhF sequences. Am. J. Bot..

[56] Hadiah J.T., Conn B.J., Quinn C.J. (2008). Infra-familial phylogeny of Urticaceae, using chloroplast sequence data. Aust. Syst. Bot..

[57] Dong W., Xu C., Li C., Sun J., Zuo Y., Shi S., Cheng T., Guo J., Zhou S. (2015). ycf1, the most promising plastid DNA barcode of land plants. Sci. Rep..

[58] Li J., Tang J., Zeng S., Han F., Yuan J., Yu J. (2021). Comparative plastid genomics of four Pilea (Urticaceae) species: Insight into interspecific plastid genome diversity in Pilea. BMC Plant Biol..

[59] Yan M., Fritsch P.W., Moore M.J., Feng T., Meng A., Yang J., Deng T., Zhao C., Yao X., Sun H. (2018). Plastid phylogenomics resolves infrafamilial relationships of the Styracaceae and sheds light on the backbone relationships of the Ericales. Mol. Phylogenet. Evol..

[60] Xue S., Shi T., Luo W., Ni X., Iqbal S., Ni Z., Huang X., Yao D., Shen Z., Gao Z. (2019). Comparative analysis of the complete chloroplast genome among Prunus mume, P. armeniaca, and P. salicina. Hortic. Res..

[61] Qi W.H., Jiang X.M., Yan C.C., Zhang W.Q., Xiao G.S., Yue B.S., Zhou C.Q. (2018). Distribution patterns and variation analysis of simple sequence repeats in different genomic regions of bovid genomes. Sci. Rep..

[62] Wang H., Jiang J., Chen S., Qi X., Peng H., Li P., Song A., Guan Z., Fang W., Liao Y. (2013). Next-generation sequencing of the Chrysanthemum nankingense (Asteraceae) transcriptome permits large-scale unigene assembly and SSR marker discovery. PLoS ONE.

[63] Zhang X., Rong C., Qin L., Mo C., Fan L., Yan J., Zhang M. (2018). Complete Chloroplast Genome Sequence of Malus hupehensis: Genome Structure, Comparative Analysis, and Phylogenetic Relationships. Molecules.

[64] Cheng H., Li J., Zhang H., Cai B., Gao Z., Qiao Y., Mi L. (2017). The complete chloroplast genome sequence of strawberry (Fragaria × ananassa Duch.) and comparison with related species of Rosaceae. Peer. J..

[65] Hishamuddin M.S., Lee S.Y., Ng W.L., Ramlee S.I., Lamasudin D.U., Mohamed R. (2020). Comparison of eight complete chloroplast genomes of the endangered Aquilaria tree species (Thymelaeaceae) and their phylogenetic relationships. Sci. Rep..

[66] Huang L.S., Sun Y.Q., Jin Y., Gao Q., Hu X.G., Gao F.L., Yang X.L., Zhu J.J., El-Kassaby Y.A., Mao J.F. (2018). Development of high transferability cpSSR markers for individual identification and genetic investigation in Cupressaceae species. Ecol. Evol..

[67] Alzahrani D., Albokhari E., Yaradua S., Abba A. (2021). Complete chloroplast genome sequences of Dipterygium glaucum and Cleome chrysantha and other Cleomaceae Species, comparative analysis and phylogenetic relationships. Saudi J. Biol. Sci..

[68] Bondar E.I., Troukhan M.E., Krutovsky K.V., Tatarinova T.V. (2022). Genome-Wide Prediction of Transcription Start Sites in Conifers. Int. J. Mol. Sci..

[69] Wang R.-N., Milne R.I., Du X.-Y., Liu J., Wu Z.-Y. (2020). Characteristics and mutational hotspots of plastomes in Debregeasia (Urticaceae). Front. Genet..

[70] Li Y., Dong Y., Liu Y., Yu X., Yang M., Huang Y. (2021). Comparative analyses of Euonymus chloroplast genomes: Genetic structure, screening for loci with suitable polymorphism, positive selection genes, and phylogenetic relationships within Celastrineae. Front. Plant Sci..

[71] Menges E.S. (1987). Biomass Allocation and Geometry of the Clonal Forest Herb Laportea canadensis: Adaptive Responses to the Environment or Allometric Constraints?. Am. J. Bot..

[72] Dong W., Liu J., Yu J., Wang L., Zhou S. (2012). Highly variable chloroplast markers for evaluating plant phylogeny at low taxonomic levels and for DNA barcoding. PLoS ONE.

[73] Bhellum B.L., Singh B. (2016). A new species of Laportea Gaudich.(Urticaceae) from Himalaya, India. Bangladesh J. Plant Taxon..

[74] Sun K., Sun Q., Xu W., Chen C., Wang B., Wang Y. (2022). The complete chloroplast genome of Laportea bulbifera (Sieb. et Zucc.) Wedd. and its phylogenetic analysis. Mitochondrial DNA Part B.

