# Supplementary material for: The Complete Chloroplast Genome Sequence of Laportea bulbifera (Sieb. et Zucc.) Wedd. and Comparative Analysis with Its Congeneric Species
Source: Genes (Basel). 2022 Nov 28;13(12):2230. doi: 10.3390/genes13122230 (PMC9778553; doi:10.3390/genes13122230)

Supplementary material

Supplementary table

Table S1. Chloroplast genomes are scattered in repeat sequence characteristic values

Repeat unit one length （bp）	Repeat Type	The first point of repeat unit	Repeat unit two length （bp）	The second point of repeat unit	Repeat unit interval	E-value
38	P	165	38	165	0	8.38E-14
37	F	42832	37	96316	0	3.35E-13
37	P	42832	37	136066	0	3.35E-13
32	P	6628	32	6628	0	3.43E-10
30	P	7834	30	44273	0	5.49E-09
28	P	28811	28	28844	0	8.78E-08
25	P	206	25	235	0	5.62E-06
23	F	42832	23	117139	0	8.99E-05
23	F	96316	23	117139	0	8.99E-05
23	P	117139	23	136080	0	8.99E-05
22	F	68423	22	120797	0	3.60E-04
21	F	7840	21	34612	0	1.44E-03
21	P	34612	21	44276	0	1.44E-03
21	F	66244	21	66265	0	1.44E-03
21	F	89671	21	89689	0	1.44E-03
21	P	89671	21	142709	0	1.44E-03
21	P	89689	21	142727	0	1.44E-03
21	F	142709	21	142727	0	1.44E-03
20	P	34680	20	44215	0	5.76E-03
20	P	35391	20	35421	0	5.76E-03
20	P	42132	20	42132	0	5.76E-03
20	R	49468	20	49468	0	5.76E-03
19	F	4636	19	22294	0	2.30E-02
19	F	9319	19	35677	0	2.30E-02
19	F	34798	19	106210	0	2.30E-02
19	P	34798	19	126190	0	2.30E-02
19	R	78081	19	78081	0	2.30E-02
19	R	124094	19	124094	0	2.30E-02
18	P	2833	18	2833	0	9.21E-02
18	R	7469	18	65507	0	9.21E-02
18	F	17721	18	45666	0	9.21E-02
18	P	18192	18	18192	0	9.21E-02
18	R	30408	18	80670	0	9.21E-02
18	P	31084	18	64059	0	9.21E-02
18	C	35541	18	44113	0	9.21E-02

18	C	44122	18	109609	0	9.21E-02
18	F	47432	18	47496	0	9.21E-02
18	R	57809	18	64053	0	9.21E-02
18	R	69674	18	80669	0	9.21E-02
18	F	80801	18	110252	0	9.21E-02
18	P	99684	18	105958	0	9.21E-02
18	F	99684	18	126443	0	9.21E-02
18	F	105958	18	132717	0	9.21E-02
18	C	111866	18	122668	0	9.21E-02
18	R	124256	18	124256	0	9.21E-02
18	P	126443	18	132717	0	9.21E-02
17	R	6197	17	6197	0	3.68E-01
17	R	8304	17	8304	0	3.68E-01
17	P	8760	17	17714	0	3.68E-01

Note： P = palindromic repeat, F = forward repeat, R = reverse repeat, and C = complement repeat.

Table S2. Codon Usage in this chloroplast genome

Codon	Amino acid	Frequency	Number
GCA	A	14.191	635
GCC	A	8.135	364
GCG	A	6.66	298
GCT	A	23.645	1058
TGC	C	3.241	145
TGT	C	9.208	412
GAC	D	7.978	357
GAT	D	30.662	1372
GAA	E	38.573	1726
GAG	E	12.113	542
TTC	F	18.102	810
TTT	F	41.546	1859
GGA	G	24.896	1114
GGC	G	8.448	378
GGG	G	11.778	527
GGT	G	21.432	959
CAC	H	5.766	258
CAT	H	18.817	842
ATA	I	27.556	1233
ATC	I	15.219	681
ATT	I	40.92	1831
AAA	K	43.356	1940
AAG	K	12.359	553
CTA	L	13.565	607
CTC	L	6.995	313
CTG	L	6.928	310

CTT	L	22.773	1019
TTA	L	34.461	1542
TTG	L	21.253	951
ATG	M	22.639	1013
AAC	N	10.861	486
AAT	N	37.88	1695
CCA	P	11.398	510
CCC	P	9.297	416
CCG	P	5.699	255
CCT	P	14.661	656
CAA	Q	27.757	1242
CAG	Q	8.023	359
AGA	R	17.231	771
AGG	R	7.397	331
CGA	R	13.633	610
CGC	R	4.403	197
CGG	R	4.805	215
CGT	R	12.448	557
AGC	S	5.23	234
AGT	S	14.795	662
TCA	S	14.705	658
TCC	S	11.509	515
TCG	S	6.213	278
TCT	S	21.454	960
ACA	T	16.18	724
ACC	T	7.8	349
ACG	T	5.565	249
ACT	T	21.142	946
GTA	V	19.465	871
GTC	V	5.922	265
GTG	V	7.554	338
GTT	V	20.806	931
TGG	W	17.432	780
TAC	Y	7.487	335
TAT	Y	30.662	1372
TAA	*	3.039	136
TAG	*	2.078	93
TGA	*	2.257	101

Table S3. Predicted RNA editing site in *L.bubifera* chloroplast genome

Gene	Nt Pos	AA Pos	Align Col	Effect	Score
*acc*D (C = 0.8)	821	274	303	GCC (A) => GTC (V)	0.8
	1388	463	504	CCT (P) => CTT (L)	1
	1460	487	528	ACC (T) => ATC (I)	1
*atp*A (C = 0.8)	791	264	264	CCC (P) => CTC (L)	1

*atp*B (C = 0.8)	29	10	12	GCT (A) => GTT (V)	0.86
	31	11	13	CCC (P) => TCC (S)	1
*atp*F (C = 0.8)	92	31	31	CCA (P) => CTA (L)	0.86
*clp*P (C = 0.8)	559	187	187	CAC (H) => TAC (Y)	1
*mat*K (C = 0.8)	52	18	18	CTC (L) => TTC (F)	1
*ndh*A (C =0.8)	658	220	233	CAT (H) => TAT (Y)	1
	853	285	298	CAT (H) => TAT (Y)	1
	1205	402	415	TCA (S) => TTA (L)	0.86
	68	23	23	GCT (A) => GTT (V)	0.8
	107	36	36	CCT (P) => CTT (L)	1
	566	189	189	TCA (S) => TTA (L)	1
	853	285	285	CAC (H) => TAC (Y)	1
	1079	360	360	TCT (S) => TTT (F)	1
*ndh*B (C = 0.8)	149	50	50	TCA (S) => TTA (L)	1
	467	156	156	CCA (P) => CTA (L)	1
	542	181	181	ACG (T) => ATG (M)	1
	586	196	196	CAT (H) => TAT (Y)	1
	611	204	204	TCA (S) => TTA (L)	0.8
	737	246	246	CCA (P) => CTA (L)	1
	746	249	249	TCT (S) => TTT (F)	1
	830	277	277	TCA (S) => TTA (L)	1
	836	279	279	TCA (S) => TTA (L)	1
	1112	371	371	TCA (S) => TTA (L)	1
	1255	419	419	CAT (H) => TAT (Y)	1
	1481	494	494	CCA (P) => CTA (L)	1
*ndh*D (C = 0.8)	29	10	10	ACG (T) => ATG (M)	1
	340	114	114	CGG (R) => TGG (W)	0.8
	410	137	137	CCA (P) => CTA (L)	1
	626	209	209	TCA (S) => TTA (L)	1
	701	234	234	TCA (S) => TTA (L)	1
	905	302	302	TCA (S) => TTA (L)	1
	914	305	305	CCT (P) => CTT (L)	1
	1325	442	442	TCA (S) => TTA (L)	0.8
	1432	478	478	CTT (L) => TTT (F)	0.8
*ndh*F (C = 0.8)	196	66	66	CCT (P) => TCT (S)	1
	1427	476	476	ACA (T) => ATA (I)	0.8
*ndh*G (C = 0.8)	314	105	105	ACA (T) => ATA (I)	0.8
	494	165	165	GCC (A) => GTC (V)	0.8
*psb*E (C = 0.8)	214	72	72	CCT (P) => TCT (S)	1
*psb*F (C = 0.8)	77	26	26	TCT (S) => TTT (F)	1
*rpl*2 (C = 0.8)	547	183	186	CCC (P) => TCC (S)	1
*rpo*A (C = 0.8)	830	277	279	TCA (S) => TTA (L)	1
*rpo*C1 (C =0.8)	41	14	14	TCG (S) => TTG (L)	1
	191	64	64	ACA (T) => ATA (I)	1

	1550	517	534	ACG (T) => ATG (M)	0.86
*rps*2 (C = 0.8)	248	83	83	TCA (S) => TTA (L)	1
*rps*14 (C = 0.8)	80	27	27	TCA (S) => TTA (L)	1
	149	50	53	CCA (P) => CTA (L)	1
*rps*16 (C = 0.8)	212	71	71	TCA (S) => TTA (L)	0.83

Supplementary figure

Figure S1. Global alignment of eight chloroplast genomes of *Laportea* using mVISTA. Y-axis indicates the range of identity (50~100%). Alignment was performed using *L. aestuans* as a reference.

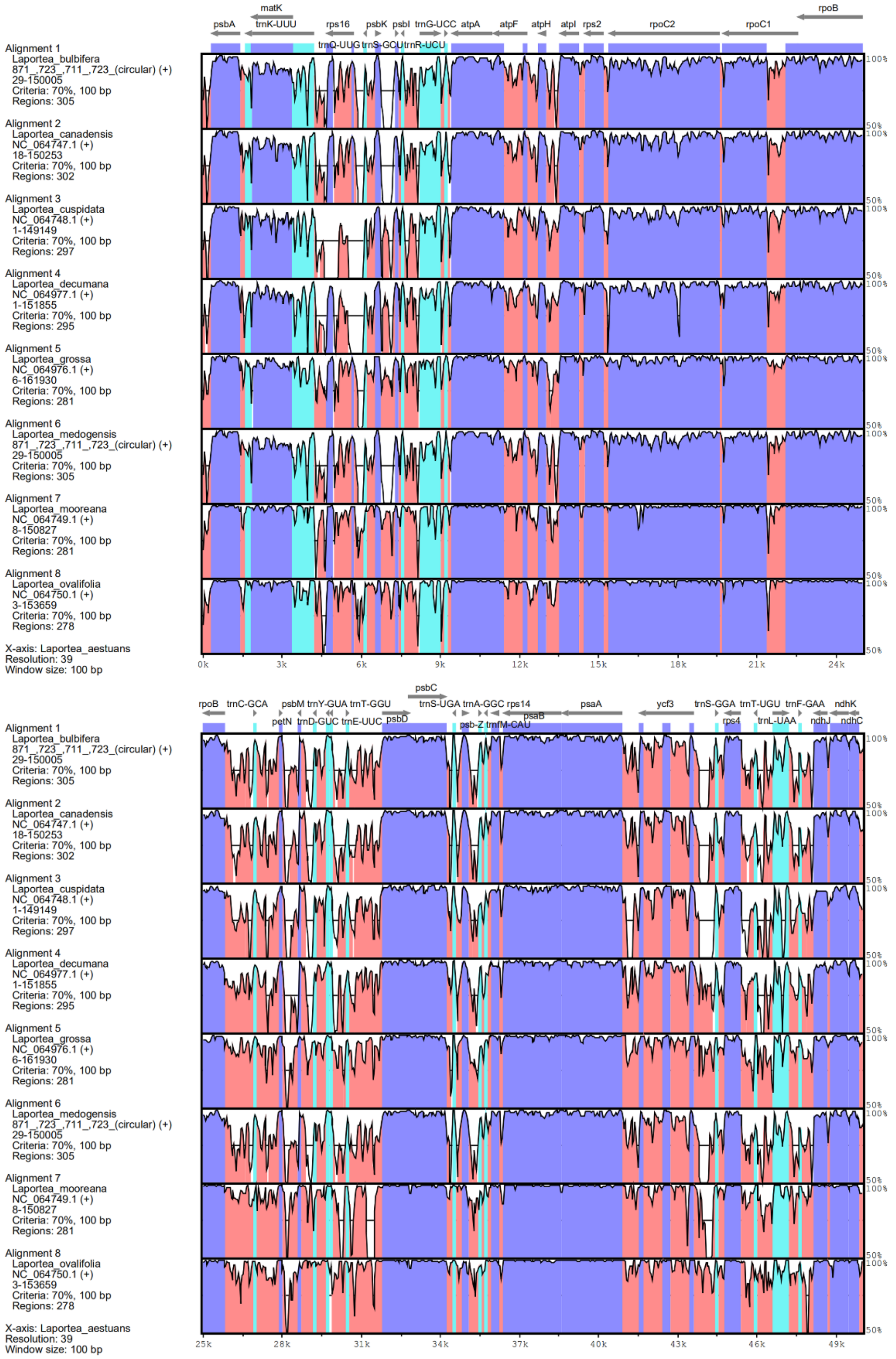

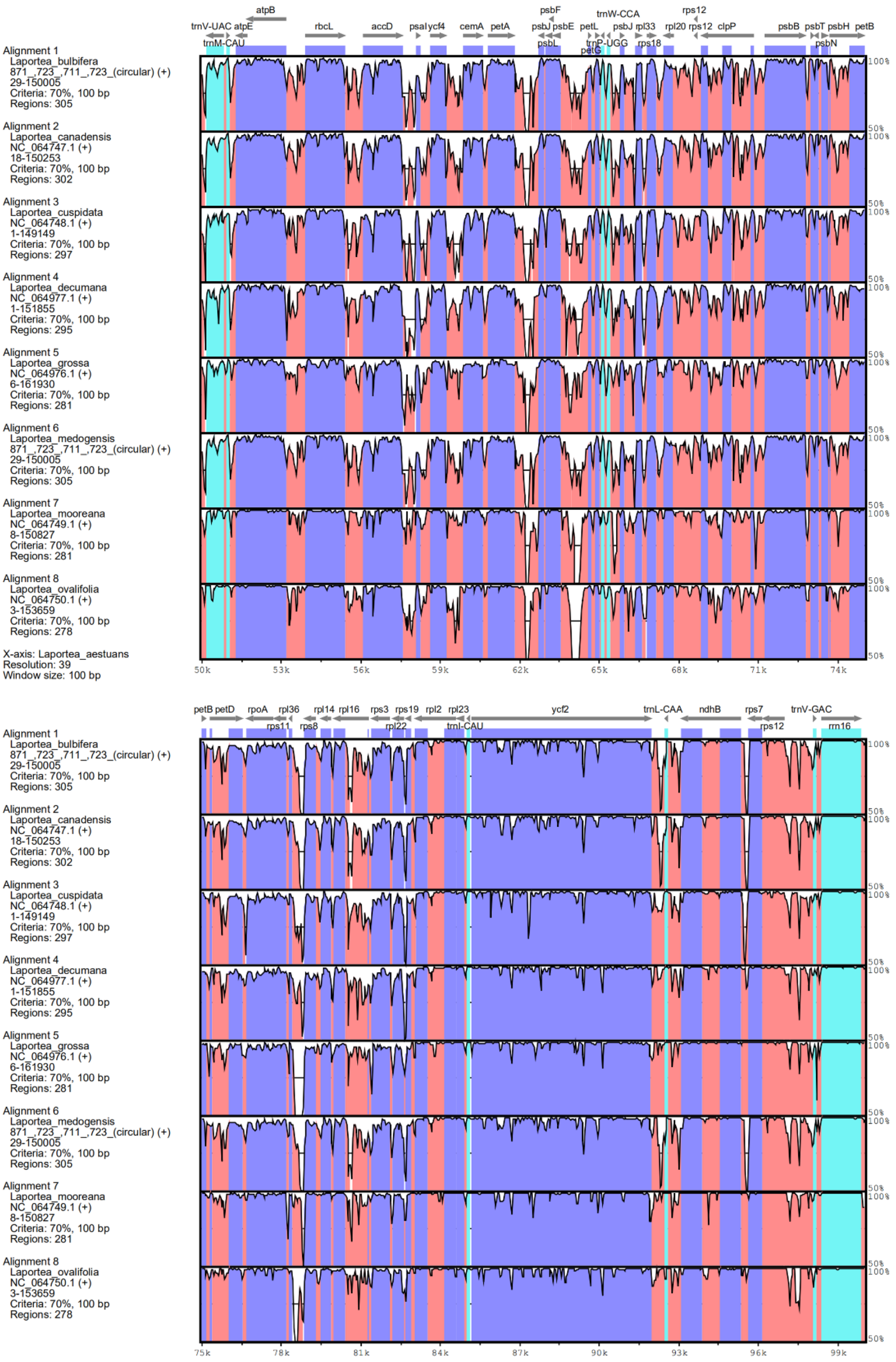
atpB
psbF
trnW-CCA
rps12
trnV-UAC atpE
rbcL
accD
psaI ycf4
cemA
petA
psbJ psbE
petL
psbJ rpl33
rpl20 rps12
clpP
psbB
psbT psbH
petB
trnM-CAU
psbL
petG
trnP-UGG
rps18
psbN
Alignment 1
Laportea_bulbifera
871_,723_,711_,723_(circular) (+)
29-150005
Criteria: 70%, 100 bp
Regions: 305
Alignment 2
Laportea_canadensis
NC_064747.1 (+)
18-150253
Criteria: 70%, 100 bp
Regions: 302
Alignment 3
Laportea_cuspidata
NC_064748.1 (+)
1-149149
Criteria: 70%, 100 bp
Regions: 297
Alignment 4
Laportea_decumana
NC_064977.1 (+)
1-151855
Criteria: 70%, 100 bp
Regions: 295
Alignment 5
Laportea_grossa
NC_064976.1 (+)
6-161930
Criteria: 70%, 100 bp
Regions: 281
Alignment 6
Laportea_medogensis
871_,723_,711_,723_(circular) (+)
29-150005
Criteria: 70%, 100 bp
Regions: 305
Alignment 7
Laportea_mooreana
NC_064749.1 (+)
8-150827
Criteria: 70%, 100 bp
Regions: 281
Alignment 8
Laportea_ovalifolia
NC_064750.1 (+)
3-153659
Criteria: 70%, 100 bp
Regions: 278
X-axis: Laportea_aestuans
Resolution: 39
Window size: 100 bp
100%
50%
50k 53k 56k 59k 62k 65k 68k 71k 74k
petB petD
rpoA
rpl36
rpl14 rpl16
rps3
rps19
rpl2
rpl23
ycf2
trnL-CAA
ndhB
rps7
trnV-GAC
rps11
rps8
rpl22
trnI-CAU
rps12
rrn16
75k 78k 81k 84k 87k 90k 93k 96k 99k

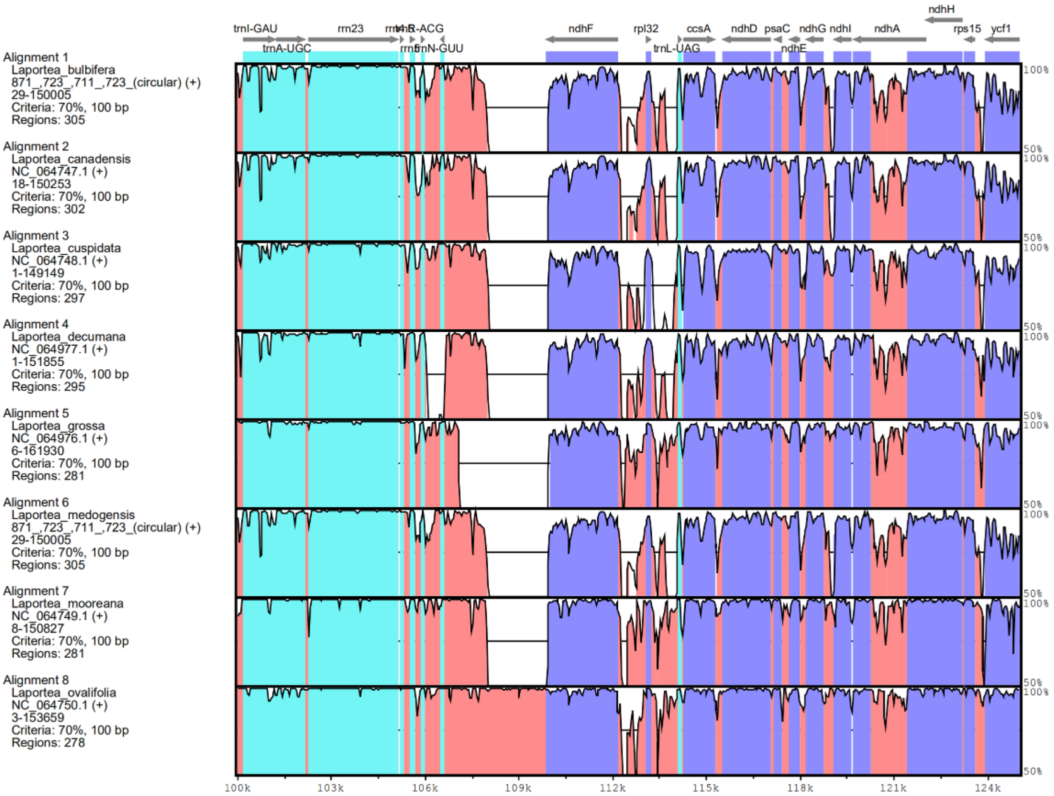
Alignment 1
Laportea_bulbifera
871_,723_,711_,723_(circular) (+)
29-150005
Criteria: 70%, 100 bp
Regions: 305
Alignment 2
Laportea_canadensis
NC_064747.1 (+)
18-150253
Criteria: 70%, 100 bp
Regions: 302
Alignment 3
Laportea_cuspidata
NC_064748.1 (+)
1-149149
Criteria: 70%, 100 bp
Regions: 297
Alignment 4
Laportea_decumana
NC_064977.1 (+)
1-151855
Criteria: 70%, 100 bp
Regions: 295
Alignment 5
Laportea_grossa
NC_064976.1 (+)
6-161930
Criteria: 70%, 100 bp
Regions: 281
Alignment 6
Laportea_medogensis
871_,723_,711_,723_(circular) (+)
29-150005
Criteria: 70%, 100 bp
Regions: 305
Alignment 7
Laportea_mooreana
NC_064749.1 (+)
8-150827
Criteria: 70%, 100 bp
Regions: 281
Alignment 8
Laportea_ovalifolia
NC_064750.1 (+)
3-153659
Criteria: 70%, 100 bp
Regions: 278
trnI-GAU
trnA-UGC
rrn23
trnR-ACG
trnN-GUU
ndhF
rpl32
trnL-UAG
ccsA
ndhD
psaC
ndhE
ndhG
ndhI
ndhA
ndhH
rps15
ycf1
100%
50%
100k
103k
106k
109k
112k
115k
118k
121k
124k

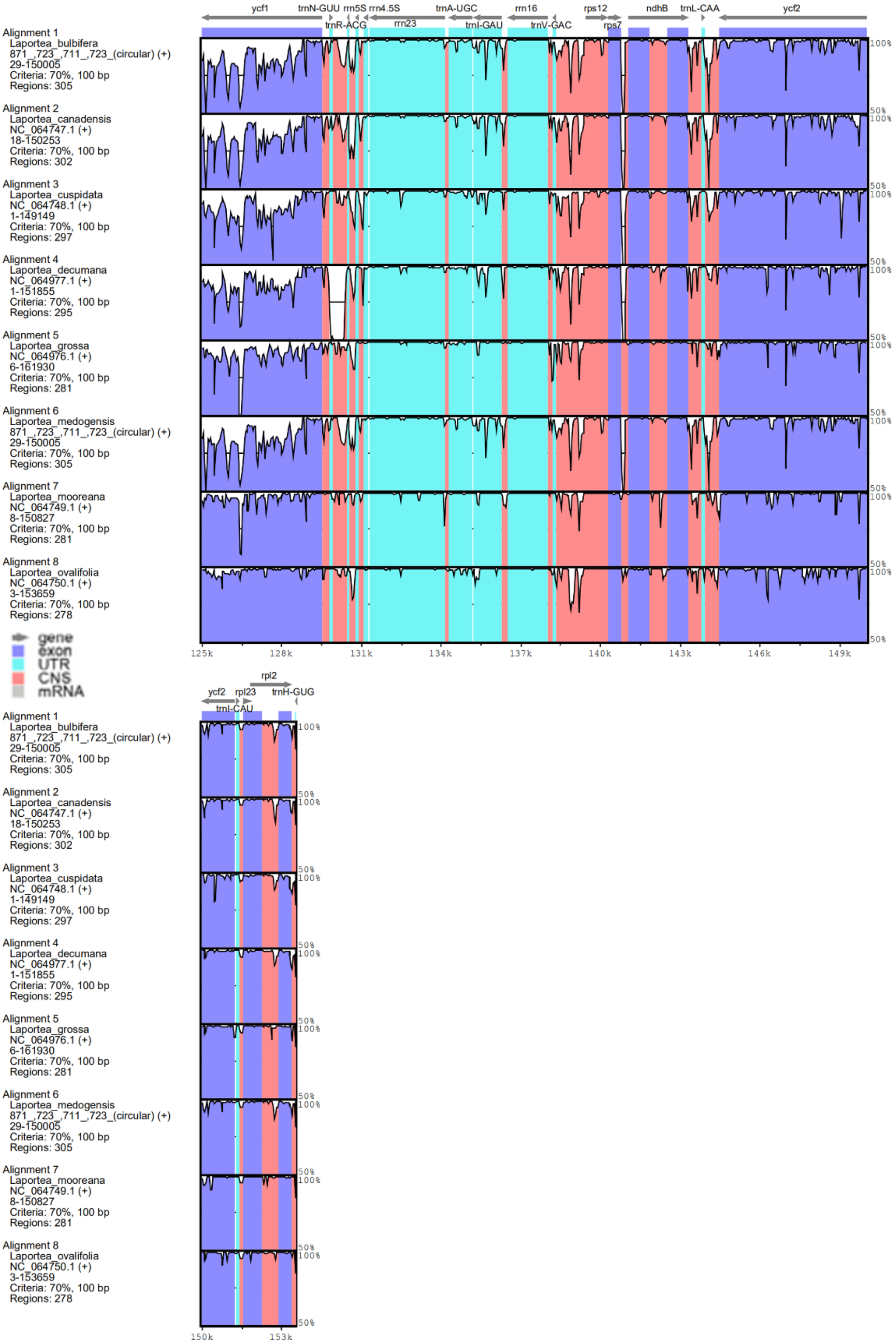
ycf1
trnN-GUU
rrn5S
rrn4.5S
trnR-ACG
rrn23
trnA-UGC
trnI-GAU
rrn16
trnV-GAC
rps12
rps7
ndhB
trnL-CAA
ycf2
Alignment 1
Laportea_bulbifera
871_,723_,711_,723_(circular) (+)
29-150005
Criteria: 70%, 100 bp
Regions: 305
Alignment 2
Laportea_canadensis
NC_064747.1 (+)
18-150253
Criteria: 70%, 100 bp
Regions: 302
Alignment 3
Laportea_cuspidata
NC_064748.1 (+)
1-149149
Criteria: 70%, 100 bp
Regions: 297
Alignment 4
Laportea_decumana
NC_064977.1 (+)
1-151855
Criteria: 70%, 100 bp
Regions: 295
Alignment 5
Laportea_grossa
NC_064976.1 (+)
6-161930
Criteria: 70%, 100 bp
Regions: 281
Alignment 6
Laportea_medogensis
871_,723_,711_,723_(circular) (+)
29-150005
Criteria: 70%, 100 bp
Regions: 305
Alignment 7
Laportea_mooreana
NC_064749.1 (+)
8-150827
Criteria: 70%, 100 bp
Regions: 281
Alignment 8
Laportea_ovalifolia
NC_064750.1 (+)
3-153659
Criteria: 70%, 100 bp
Regions: 278
100%
50%
125k
128k
131k
134k
137k
140k
143k
146k
149k
gene
exon
UTR
CNS
mRNA
rpl2
ycf2
rpl23
trnH-GUG
trnI-CAU
150k
153k

Figure S2. The dN/dS values between each plastid gene in the *Laportera* species are shown as box plots.

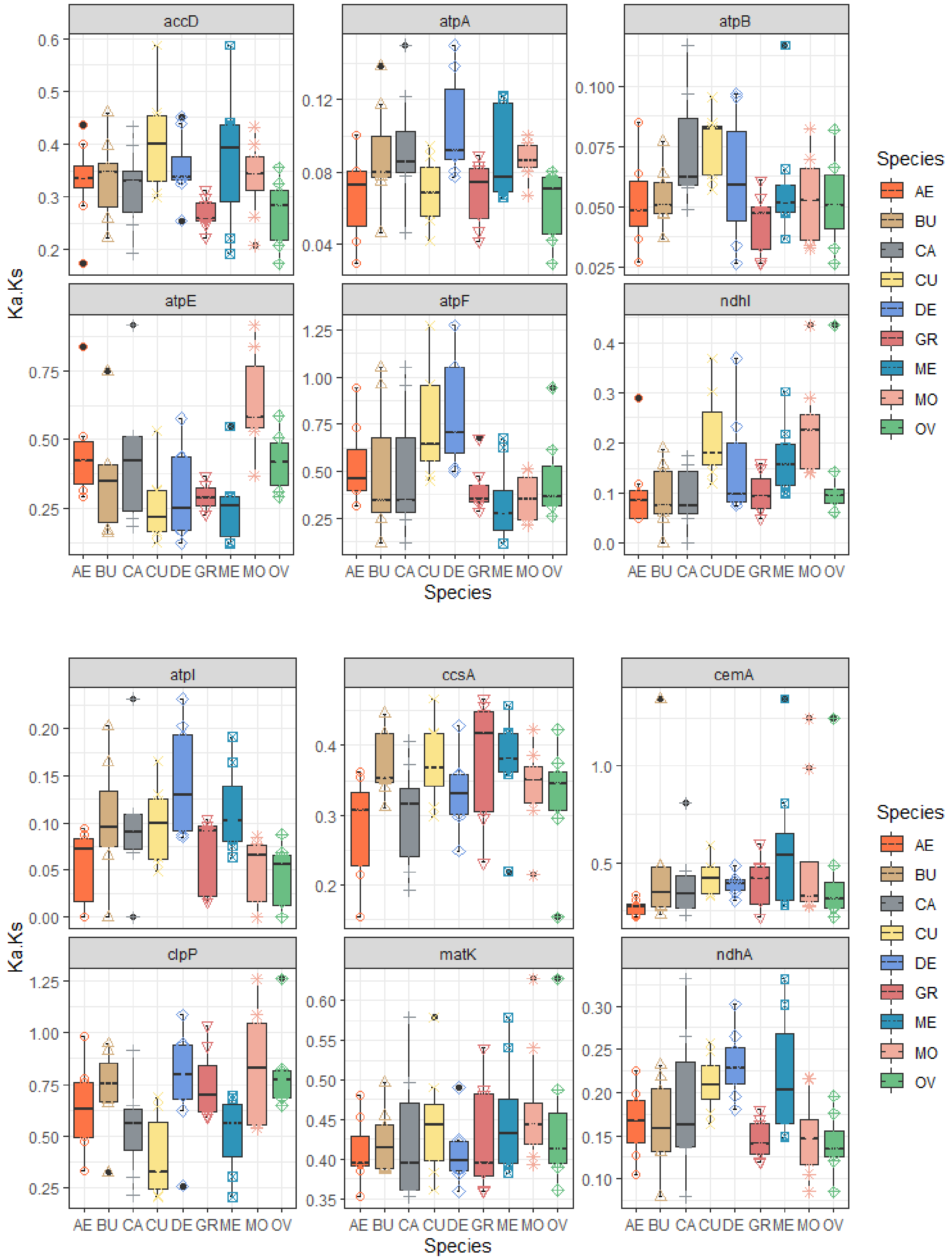

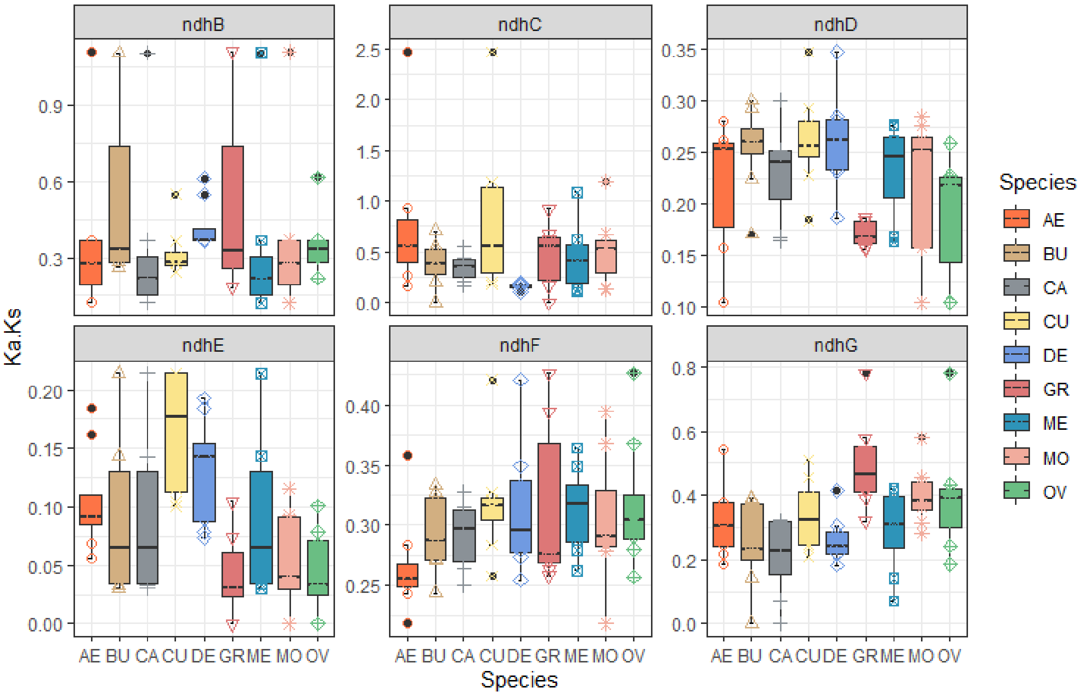
ndhB
ndhC
ndhD
ndhE
ndhF
ndhG
Ka.Ks
Species
Species
AE
BU
CA
CU
DE
GR
ME
MO
OV

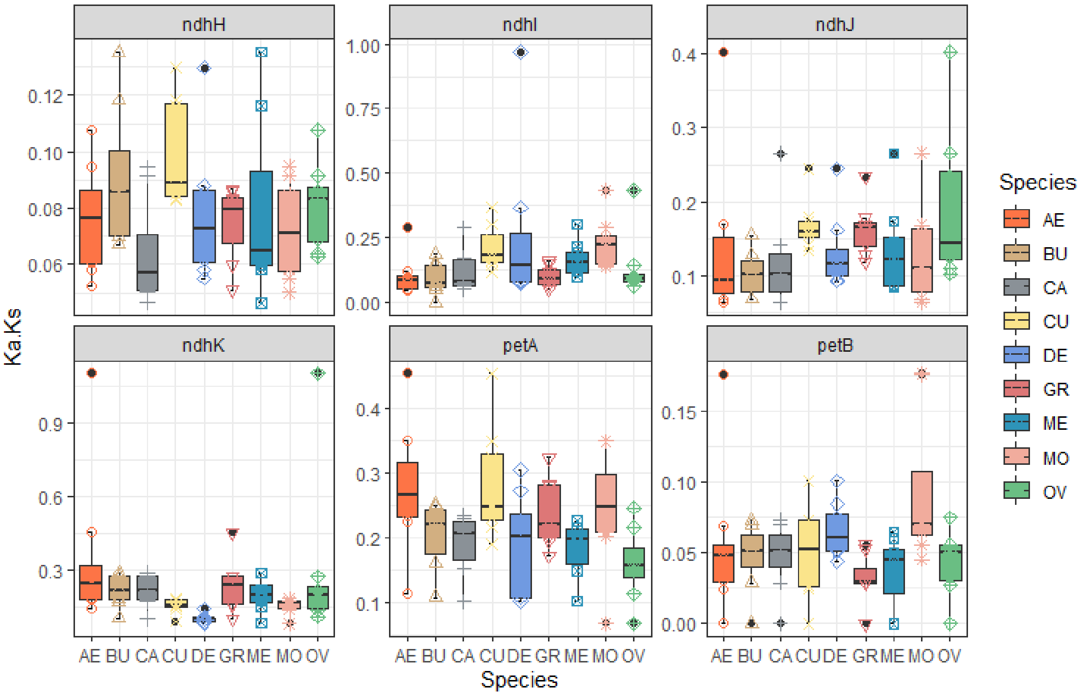
ndhH
ndhI
ndhJ
ndhK
petA
petB
Ka.Ks
Species
Species
AE
BU
CA
CU
DE
GR
ME
MO
OV

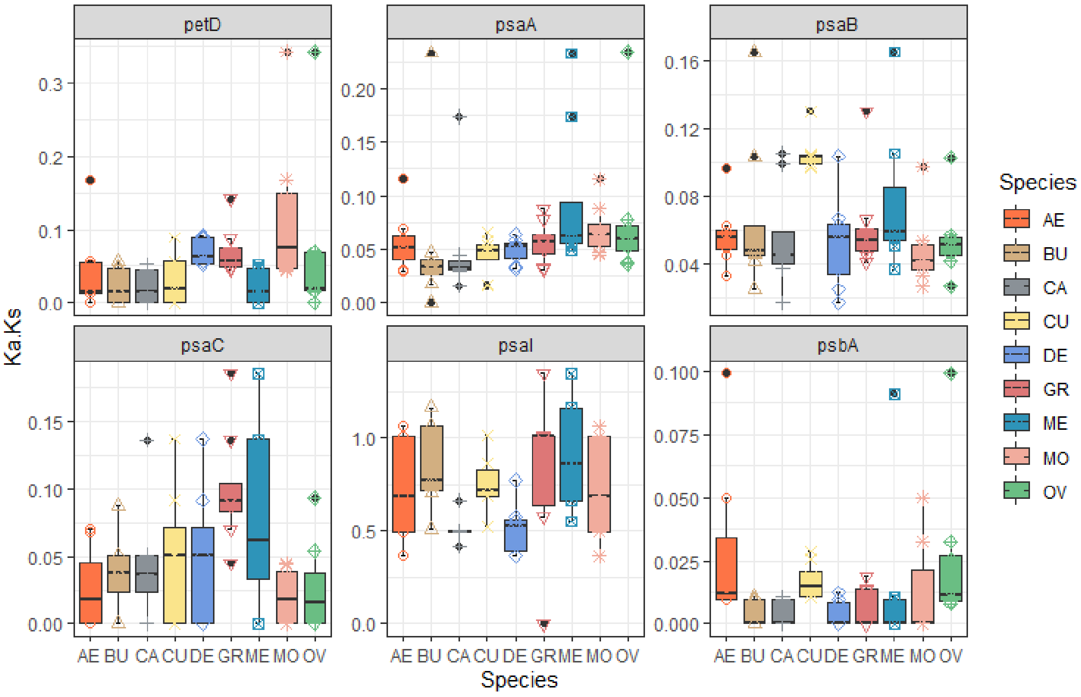
petD
psaA
psaB
psaC
psaI
psbA
Ka.Ks
Species
AE BU CA CU DE GR ME MO OV
Species
AE
BU
CA
CU
DE
GR
ME
MO
OV

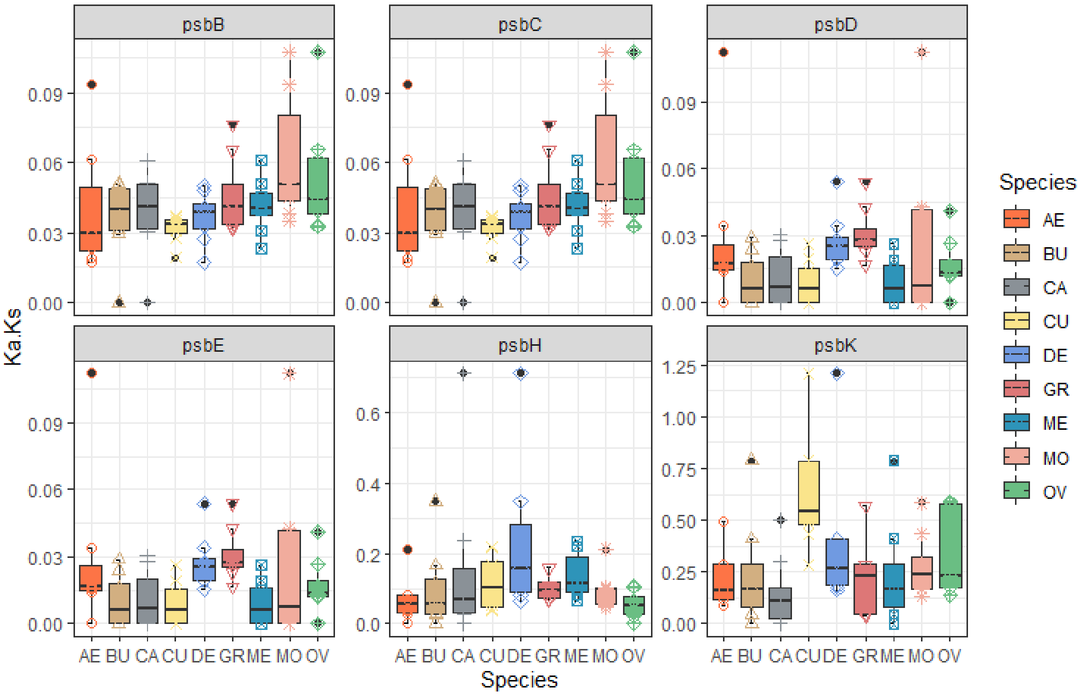
psbB
psbC
psbD
psbE
psbH
psbK
Ka.Ks
Species
AE BU CA CU DE GR ME MO OV
Species
AE
BU
CA
CU
DE
GR
ME
MO
OV

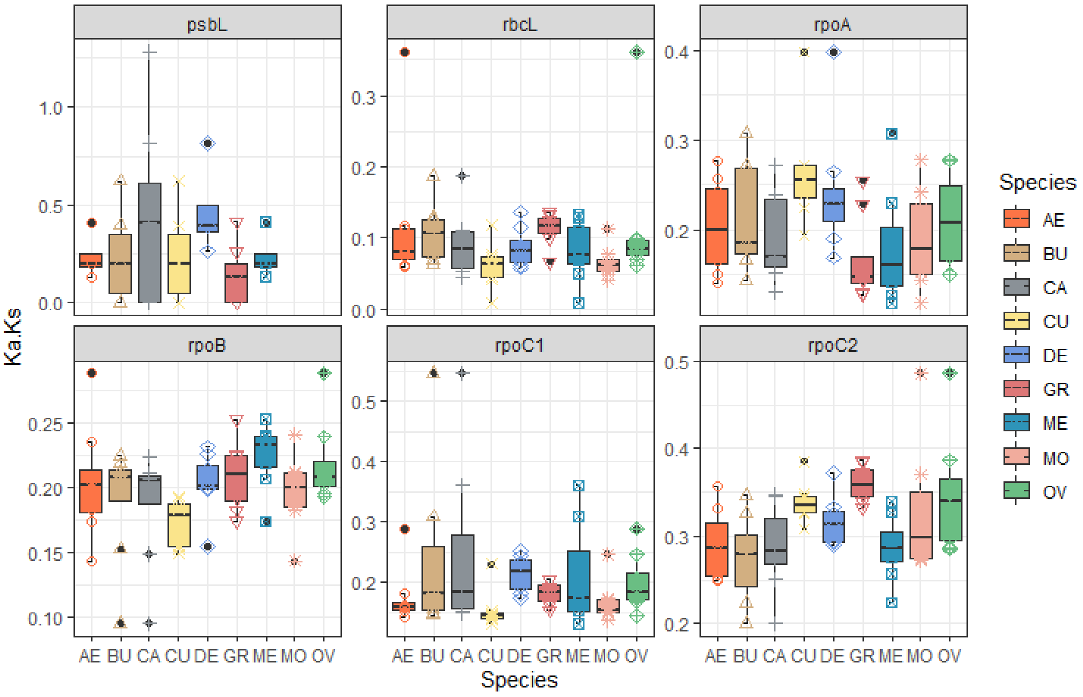
psbL
rbcL
rpoA
rpoB
rpoC1
rpoC2
Ka.Ks
Species
AE BU CA CU DE GR ME MO OV
Species
AE
BU
CA
CU
DE
GR
ME
MO
OV

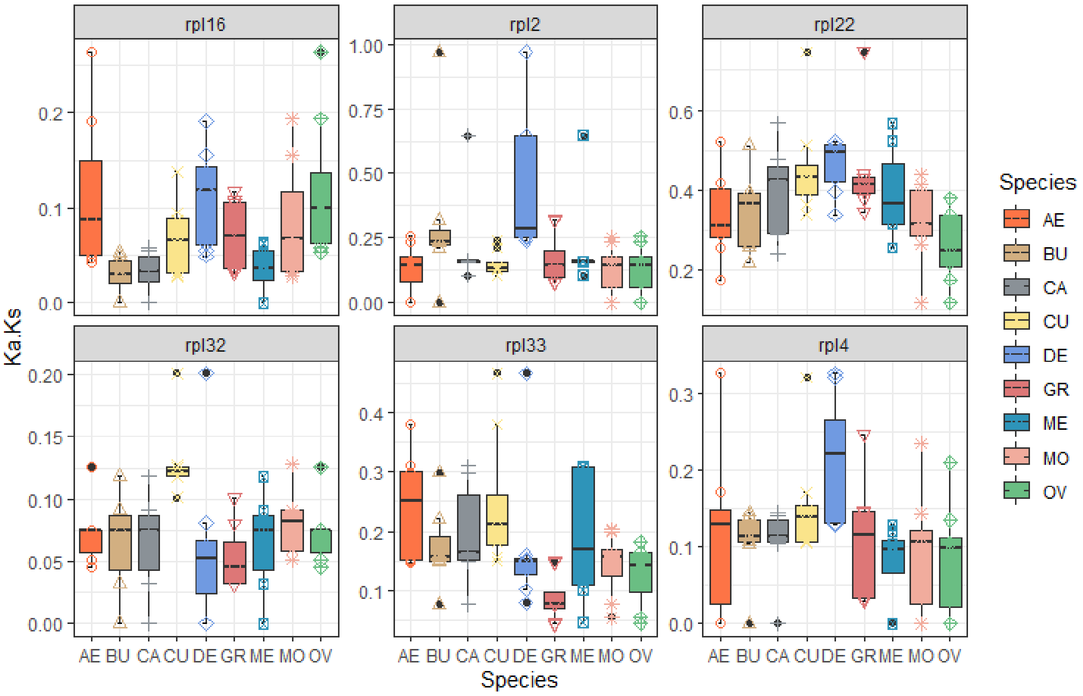
rpl16
rpl2
rpl22
rpl32
rpl33
rpl4
Ka.Ks
Species
AE BU CA CU DE GR ME MO OV
Species
AE
BU
CA
CU
DE
GR
ME
MO
OV

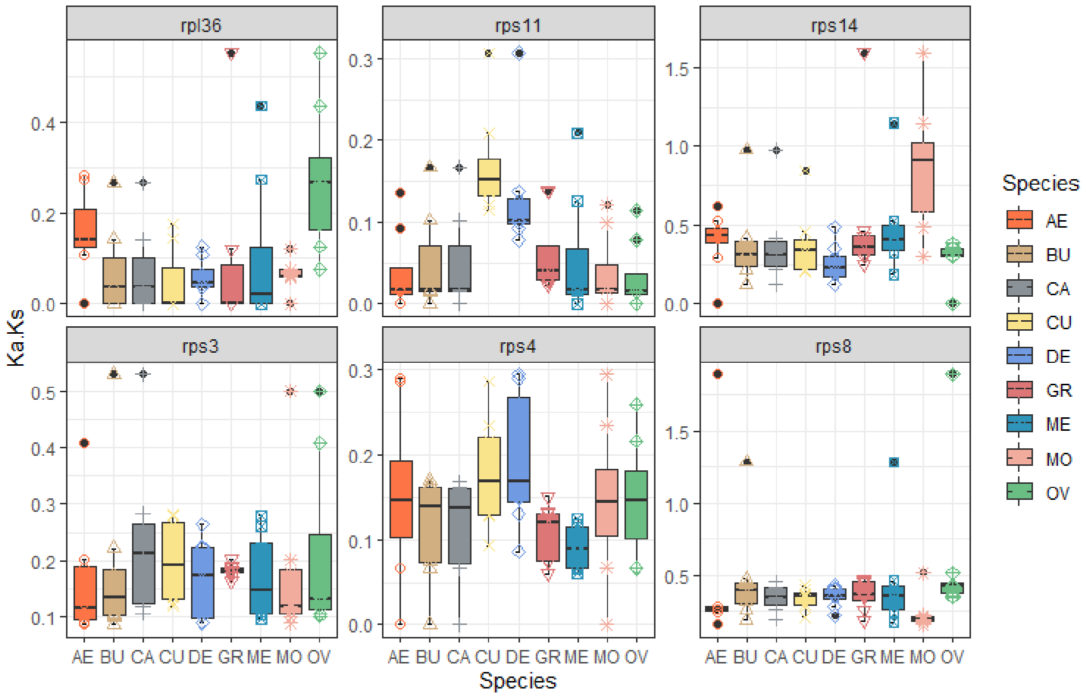
rpl36
rps11
rps14
rps3
rps4
rps8
Ka.Ks
Species
AE BU CA CU DE GR ME MO OV
Species
AE
BU
CA
CU
DE
GR
ME
MO
OV

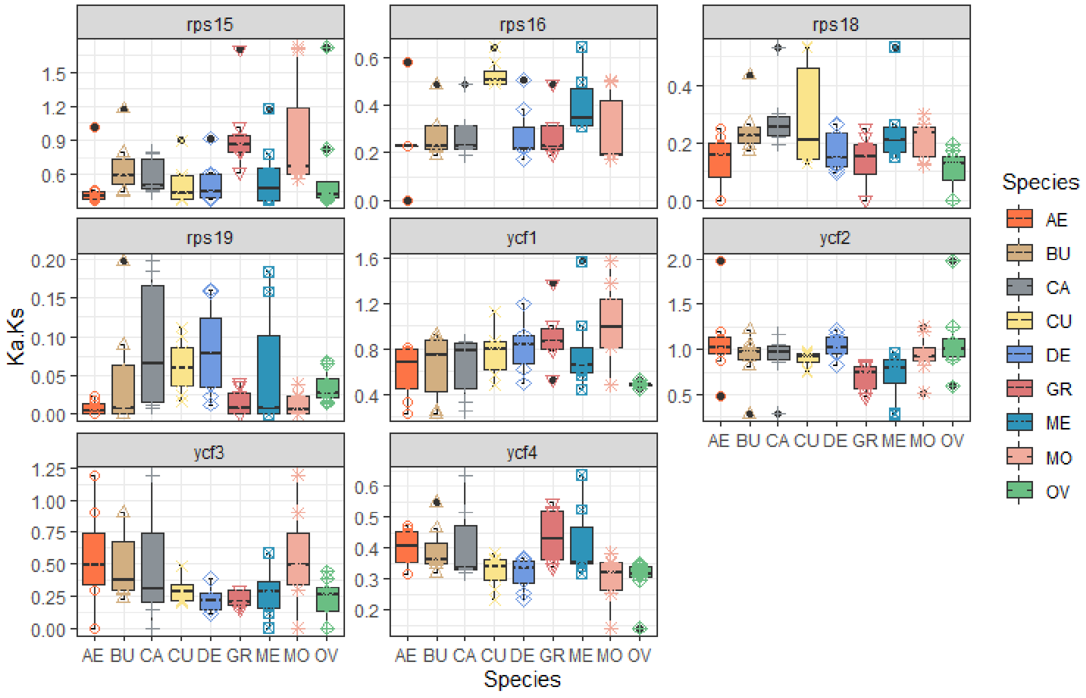
rps15
rps16
rps18
rps19
ycf1
ycf2
ycf3
ycf4
Ka.Ks
Species
AE BU CA CU DE GR ME MO OV
Species
AE
BU
CA
CU
DE
GR
ME
MO
OV

Figure S3. The phylogenetic tree of species from *Laportea* is based on 8 CDSs using the maximum likelihood (ML) method.

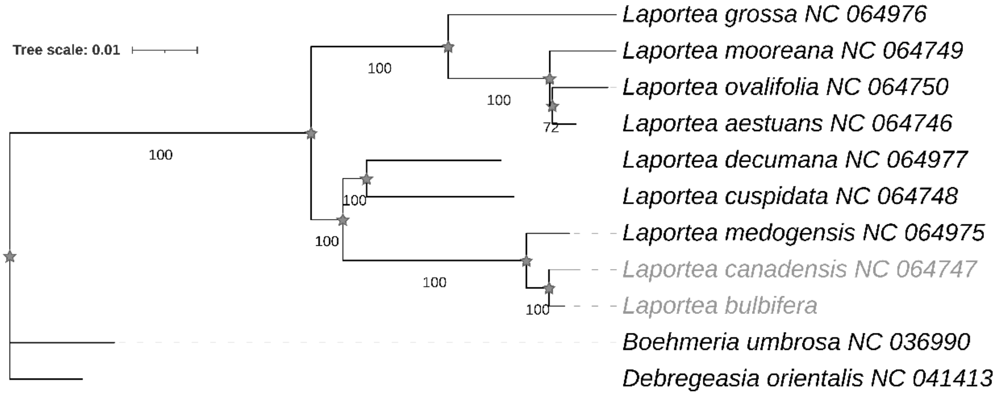

Figure S4. The phylogenetic tree of species from Urticaceae based on 115 CDSs using the test minimum evolution (ME, Figure S4a) and the unweighted pair group method with arithmetic mean (UPGMA, Figure S4b) and the neighbor-joining (NJ, Figure S4c) method.

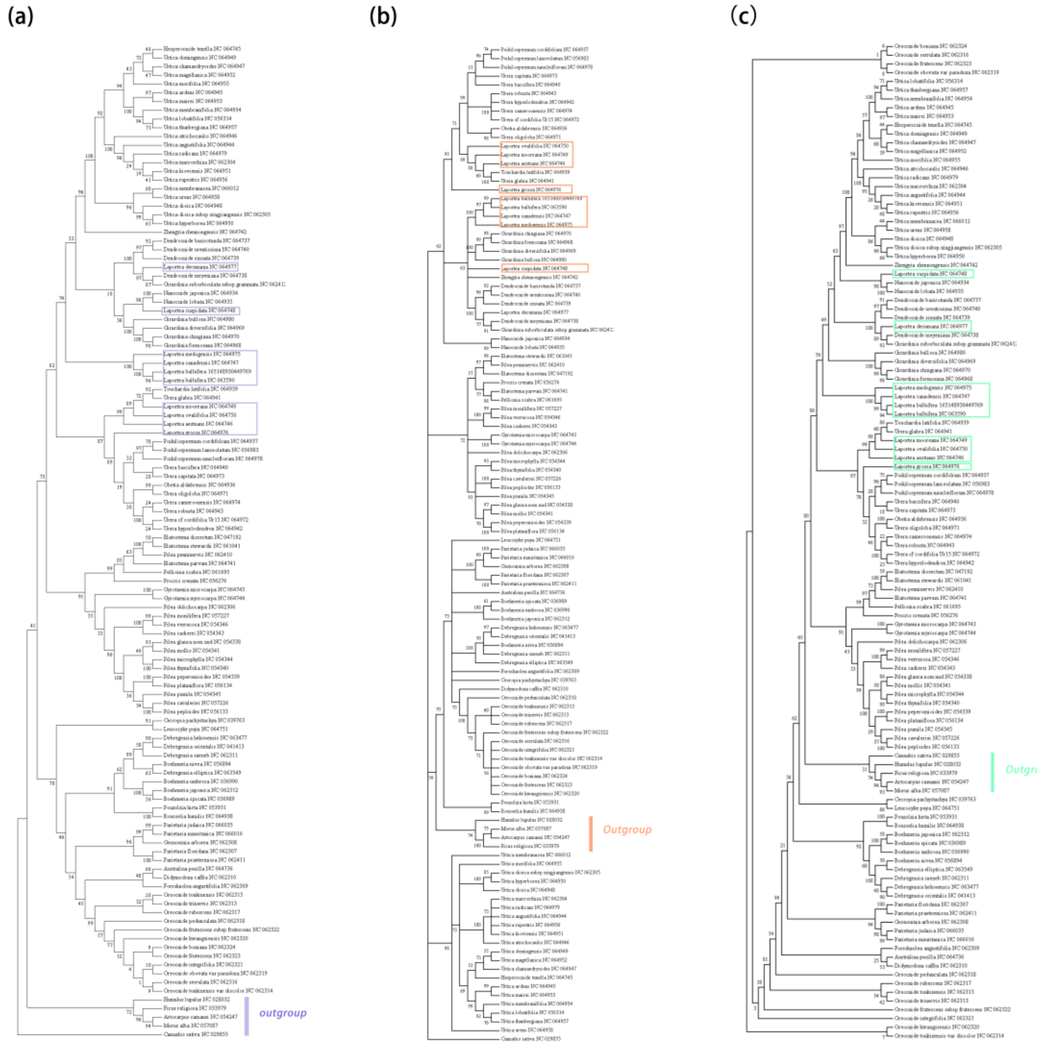

Supplement: Supplementary file 1 [file genes-13-02230-s001.zip › genes-2007688-supplementary.pdf]
